# Cationic Polystyrene-Based Hydrogels: Low-Cost and Regenerable Adsorbents to Electrostatically Remove Nitrites from Water

**DOI:** 10.3390/toxics11040312

**Published:** 2023-03-27

**Authors:** Silvana Alfei, Valentina Orlandi, Federica Grasso, Raffaella Boggia, Guendalina Zuccari

**Affiliations:** Department of Pharmacy, University of Genoa, Viale Cembrano, 16148 Genoa, Italy; valentina.orlandi@edu.unige.it (V.O.); federica.grasso@edu.unige.it (F.G.); boggia@difar.unige.it (R.B.)

**Keywords:** nitrites, metastable ions, environmental toxicity, cationic polystyrene resins (R1, R2), cationic hydrogels (R1HG, R2HG), decontamination systems, electrostatic interactions, high removal efficiency, removal by contact, removal by filtration, UV–Vis analyses, Griess reagent system (GRS), regenerable materials

## Abstract

Nitrites are metastable anions that are derived from the oxidation of ammonia by agricultural pollution, sewage, decaying protein, and other nitrogen sources. They are a recognized environmental issue due to their role in eutrophication, as well as in surface and groundwater contamination, being toxic to almost all living creatures. Recently, we reported on the high efficiency of two cationic resins (R1 and R2) forming hydrogels (R1HG and R2HG) by dispersion in water in removing anionic dyes from water by electrostatic binding. Here, aiming at developing adsorbent materials for nitrite remediation, R1, R2, R1HG, and R2HG were first tested in adsorption experiments in batches monitored by UV–Vis methods, using the Griess reagent system (GRS) in order to assess their removal efficiency by contact over time. Particularly, samples of water appositely contaminated with nitrites were analyzed by UV–Vis before and during treatment with the hydrogels. The initial concentration of nitrites was quantified (118 mg/L). Then, the removal of nitrites over time, the removal efficiency of R1HG (89.2%) and of R2HG (89.6%), their maximum adsorption (21.0 mg/g and 23.5 mg/g), as well as the adsorption kinetics and mechanisms were evaluated. Additionally, R1HG- and R2HG-based columns (h = 8–10 cm, Ø_E_ = 2 cm) mimicking mini-scale decontamination systems by filtration were used to rapidly filter samples of water polluted with nitrite that were under pressure. R1HG and R2GH were capable of totally removing nitrites (99.5% and 100%) from volumes of nitrite solutions that were 118 mg/L that is 10 times the volumes of resins used. Additionally, when extending filtration to increasing volumes of the same nitrite solution up to 60 times the volume of resins used, the removal efficiently of R1HG decreased, and that of R2HG remained stable at over 89%. Interestingly, both the worn-out hydrogels were regenerable by 1% HCl washing, without a significant reduction in their original efficiency. There is a lack of studies in the literature reporting on novel methods to remove nitrite from water. R1HG and especially R2HG represent low-cost, up-scalable, and regenerable column-packing materials with promise for applications in the treatment of drinking water contaminated by nitrites.

## 1. Introduction

Untreated or badly treated waste released into bodies of water causes water pollution, which is one of the most widespread problems afflicting people throughout the world. Polluted waters are destructive to plants and organisms living in or around the aquatic ecosystem and can also harm the people, plants, and animals that consume these waters [1]. Water pollutants mainly include pathogens, inorganic compounds, organic material, and macroscopic pollutants.

Among inorganic pollutants, inorganic nitrogen pollution, mainly in the form of reactive ions such as ammonium (NH_4_^+^), nitrite (NO_2_^−^), and nitrate (NO_3_^−^) dissolved in water, can have detrimental ecological and toxicological effects on aquatic ecosystems [2]. In a non-altered global nitrogen cycle, ammonium, nitrite, and nitrate ions are naturally removed from water by macrophytes, algae, and bacteria, which exploit them as sources of nitrogen [3]. Facultative anaerobic bacteria including *Achromobacter, Bacillus, Micrococcus,* and *Pseudomonas* can utilize nitrites and nitrates as terminal acceptors of electrons, resulting in the ultimate formation of N_2_O and N_2_ [4]. Excessively high levels of inorganic nitrogen, which the functions of ecological systems do not manage to assimilate (N-saturated ecosystems), can cause adverse effects on the least tolerant organisms and can overstimulate the growth of aquatic plants and algae. Excessive growth of these organisms can in turn clog water intake, use up dissolved oxygen as the organisms decompose, block light to deeper water, and result in killing fish due to a lack of dissolved oxygen [5].

### 1.1. Sources of Inorganic Nitrogen Pollutants

Agricultural pollution, including animal husbandry and NH_3_-based fertilizer applications, sewage, decaying protein, and other nitrogen pollution sources, are suppliers of ammonia, which is oxidized (nitrified) into nitrite (NO_2_^−^) in aerobic environments. Nitrified nitrogen is in turn further oxidized into nitrate (NO_3_^−^) by bacteria that favor nitrite [6]. Conversely, nitrate can be easily reduced to nitrite in vivo. In this regard, nitrite can be produced by *Nitrosomonas, Nitrosococcus*, and *Nitrospira* bacteria in water distribution tubes where the water contains nitrate, oxygen is low, and chloramines are used to provide disinfection. In addition to existing in the environment, nitrite and nitrate exist widely in food products, mainly in the form of sodium or potassium salts. They are added to foodstuffs as preservative additives, with the principal function of inhibiting the propagation of food-poisoning microorganisms, such as *Clostridium botulinum* [7], and of improving the color and flavor of meat products [8]. Leafy vegetables and fruits are natural sources of nitrite and nitrate, but the development of modern agriculture and the abuse of inorganic fertilizers has caused a remarkable increase in nitrite and nitrate concentrations in vegetables and fruits [9]. Further, nitrite can contaminate potable water through runoff water coming into contact with fertilizer or sewage or from groundwater coming into contact with mineral deposits [10]. Additional sources of nitrites contaminating water include improperly treated sewage or septic tank effluent, or decaying plant or animal matter. Shallow wells and improperly constructed or damaged wells are extremely susceptible to nitrite contamination [10].

### 1.2. Toxicity of Nitrites and Nitrates

As above mentioned, nitrite and nitrate represent a recognized environmental concern due to their role in acidification, eutrophication (algal blooms in lakes and ponds), and groundwater contamination [11]. The eutrophication of freshwater, estuarine, and coastal marine ecosystems can cause ecological and toxicological effects that can be either directly or indirectly related to the hyper-proliferation of primary producers. The most dramatic event in eutrophic and hypereutrophic aquatic ecosystems with low water turnover rates is a reduction in dissolved oxygen (hypoxia or anoxia) causing the extensive death of both invertebrates and fish [4]. Additionally, the lack of oxygen can also promote the development of reduced compounds, such as hydrogen sulfide, with supplementary toxic effects on aquatic animals [4]. Moreover, cyanobacteria blooms associated with eutrophication can also produce toxic compounds that are harmful to livestock and human health [10].

Thanks to water salinity, due to the presence of sodium, chloride, calcium, and other ions, seawater animals are more tolerant to the toxicity of inorganic nitrogenous compounds than freshwater animals. Scientific reports have evidenced that when ingested, nitrites and nitrates might result in mutagenicity, teratogenicity, and birth defects. Furthermore, ingested nitrite can contribute to non-Hodgkin’s lymphoma and bladder and ovarian cancers, play a role in the etiology of insulin-dependent diabetes mellitus and in the development of thyroid hypertrophy, or cause spontaneous fetal death and respiratory tract infections. A potential relationship between inorganic nitrogen pollution and human infectious diseases (malaria, cholera) has also been described. To prevent aquatic ecosystems (excluding those ecosystems with naturally high N levels) from developing acidification and eutrophication, the levels of total nitrogen (TN) should be lower than 0.5–1.0 mg/L. When present in freshwater, nitrite is known to be toxic to almost all living organisms, due to its action in decreasing the capacity of the blood to carry oxygen. In fact, nitrite can cause the irreversible conversion of hemoglobin to methemoglobin in the bloodstream, compromising the ability of hemoglobin to exchange oxygen, thus interfering with the oxygen transport system in the body [12,13]. This hazard is particularly serious for pregnant women and infants, as well as for aquatic life [14,15]. In this regard, this phenomenon is known as “blue baby syndrome” in infants, and it is referred to as “brown blood disorder” disorder in fish [16]. Infants, pregnant and nursing women, as well as elderly people should avoid consuming water with high levels of nitrite or nitrate. Moreover, nitrite can also react with secondary amines and amides in the stomach to form carcinogenic N-nitrosamines [15,17]. Due to these toxic effects, many countries have placed severe restrictions on their use in processed food products [18]. The presence of nitrite in drinking water, such as in well water, may also indicate other water quality troubles, including the presence of coliform bacteria and unsafe levels of nitrate. In good drinking water, concentrations of nitrite are usually below 0.1 mg/L [10].

### 1.3. Nitrite: A Water Pollutant That Needs to Be More Carefully Pondered

Nitrite is a metastable anion that tends to accept an additional oxygen ion in the presence of oxygen (as generally occurs in surface water), becoming nitrate. For this reason, ecologists, environmental scientists, analytical chemists, and catchment managers generally do not sufficiently consider its intrinsic impact on aquatic environments and species, though more attention has been paid to nitrates. An indirect consequence of this trend is that though the literature is rich in studies concerning methods to remove nitrate from wastewater, drinking water, ground water, surface water, lakes, rivers, and seas [19], to our knowledge, studies concerning the development of new methods to remove nitrite from water are missing. In this regard, articles exist in the literature that report on nitrite removal through its oxidation into nitrates by biological treatments, hydrogen peroxide, ozone, or other costly methods that require high safety standards for the final removal of nitrates, but no method for specifically removing nitrite has been proposed recently [20,21]. Reverse osmosis, ion exchange, electrodialysis, electrocoagulation, biological denitrification, chemical denitrification, and adsorption methods are extensively described for nitrate remediation, with adsorption being the most promising method [19,22]. Concerning this, adsorption consists of the separation of compounds from the environment into the bulk or surface of a solid or liquid phase [23]. Among other separation and purification techniques such as photo-remediation, membrane technology, ion exchange, and electrochemical separation, adsorption is superior due to its unique features, such as its low cost, ease of operation, and high efficiency [23]. Zeolites, clays, biomasses, agricultural waste, activated carbons, microorganisms, nanoparticles, and metal oxides are the most widely applied, naturally available low-cost adsorbents [24].

Currently, nitrite can be removed from drinking water by reverse osmosis, distillation, or ion exchange [10], whereas boiling, carbon adsorption filters, and standard water softeners do not remove nitrite [10]. Ion exchange filters remove nitrite and other anions by adsorbing them into anion-exchange resins. Since anion-exchange resins preferentially adsorb sulfate ions, they are less effective at removing nitrite if the sulfate concentration is high [10]. Additionally, this type of filter must be replaced or regenerated before it becomes saturated, in order to avoid sulfate ions replacing nitrite ions already adsorbed by the filter, causing nitrite concentrations in the water to increase instead of decrease. In excess amounts, nitrite causes stress and diseases in fish, suffocating them, causing skin disorders, and hampering the growth rate and the development of their organs. In order to avoid this, some expedients are proposed to maintain low nitrite levels in an aquarium environment [25]. In this regard, water conditioners and nitrite removers that are able to bind to nitrite and render nitrite ions harmless to fish have been commercialized. Additionally, the use of filters with full-colonized beneficial bacteria, which are capable of converting ammonia into nitrite and then finally into nitrate, is suggested [25].

In field-based monitoring, nitrite is an effective indicator of nitrogen pollution sources, which can linger in the environment longer than sometimes assumed. As such, low-cost, up-scalable, and regenerable efficient systems that capture nitrite from the water and do not need to convert the nitrite into nitrate for nitrate removal, thus avoiding costly operations, are urgently needed.

In this regard, the adsorbent efficiency of water-insoluble polymers and copolymers (resins) in the form ammonium salts in adsorbing anionic compounds has long been studied [26,27]. These cationic macromolecules, if properly structured, may also supply hydrogels when dispersed in water. As recently reported, hydrogels are characterized by well-defined, three-dimensional (3D) porous structures, and the presence of hydrophilic functional groups, such as protonated amine groups, confers to them a high level of hydrophilicity [27]. As adsorbents, cationic hydrogels represent an important tool to remove anionic pollutants from water because of their flexible network, which allows solutes to quickly penetrate water and form stable complexes with them [27].

Additionally, hydrogels are capable of absorbing great amounts of water and of increasing to several times their original volume, thus exposing their functional groups, which become more accessible, promoting interaction with contaminants and their sorption onto polymer chains [28,29].

In this context, CMC-g-Poly (MAA-co-Aam)/Cloisite 30B (Hyd/C30B) and poly (methacrylic acid-co-acrylamide)/Cloisite 30B nanocomposite (poly (MAA-co-Aam)/Cl30B) hydrogels have demonstrated high efficiency in adsorbing methylene blue (MB) dye from wastewater samples [30,31]. Additionally, a recent paper reported the immobilization of hybrid gold–cesium nanoparticles (Au–Cs NPs) onto a magnesium ferrite (MgFe_2_O_4_) surface and detailed the behavior of the prepared composites in N_2_ adsorption–desorption experiments [32]. Recently, we reported on the synthesis and characterization of polystyrene-based cationic resins (R1 and R2) [27,33], obtained in the form of hydrochloride salts by reverse suspension copolymerization (see Scheme S1, Section S1 in the Supplementary Materials (SM)) of monomers M1 and M2 (see Figure S1, Section S1 in Supplementary Materials) [34]. Upon dispersion into an excess of water, R1 and R2 yielded cationic hydrogels (R1HG and R2HG) without the use of any other additive or gelling agent. These were believed to possess adsorbent capacities to act against aniconic pollutants due to electrostatic interactions [27].

When tested in adsorption experiments in batches over time monitored with UV–Vis spectrometry, R1 and R2 showed rapid and high adsorption efficiency towards the methyl orange (MO) azo dye and fluorescein sodium salt (F) selected as models of anionic dyes based on electrostatic interactions (97–100%) [27]. Moreover, R1HG- and R2HG-based short columns, mimicking possible decontamination systems, were capable of totally removing (100%) MO, F, and a mixture of the two by filtration under pressure in different conditions [27].

With the aim of developing low-cost, up-scalable, durable, and regenerable adsorbent materials for nitrite remediation and of preventing water contamination by nitrogen pollutants deriving from nitrite, R1, R2, R1HG, and R2HG were tested for their capability to capture and retain nitrite both by contact and by filtration. To this end, we carried out adsorption experiments using R1, R2, R1HG, and R2HG as adsorbents materials both in batches and in columns, representative of low-scale decontamination systems. The results are reported and discussed. The kinetics mechanisms of the adsorption processes and the possibility of regenerating R1 and R2 are also investigated. Collectively, R1HG and especially R2HG proved to be low-cost, up-scalable, and regenerable adsorbent materials that are promising for rapid nitrite remediation and drinking water decontamination through simple contact or filtration. Specifically, without using currently adopted methods that are more expensive and complicated, R1HG and R2HG could be capable of reducing nitrite concentrations under the legal limit of 0.5 mg/L (0.48 mg/L for R1HG and 0.11 mg/L for R2HG) in volumes of drinking water having the maximum contamination level registered in 2023 (1.0 mg/L), which would be 50–60 times higher than the volumes of resin used. Since there are a lack of papers in the literature concerning the development of new methods to remove nitrite from water, the present study has immense relevance and is especially novel, as there are no other previously published reports to compare it to. We hope that the present work might be a pioneer for more extensive literature on the subject of nitrite remediation and inspire other scientists to perform research in the field.

## 2. Materials and Methods

### 2.1. Chemicals and Instruments

Sodium nitrite (NaNO_2_) was purchased from Merk Life Science S.r.l. (Milan, Italy), and Griess reagent was obtained from VWR International S.r.l., (Milan, Italy). All other reagents and solvents were obtained from Merk (Milan, Italy) and were purified with standard procedures. M1 and M2 were prepared and characterized as previously described [34]. R1 and R2, forming the hydrogels R1HG and R2HG in water, were prepared and characterized according to reported procedures [27,33]. The optical microscopy and SEM analyses reported in the Supplementary Materials were performed using the same instruments as recently described [25]. Lyophilization and centrifugations were performed as previously described [35]. UV–Vis analyses were carried out using an Agilent Cary 100 UV/Vis Spectrophotometer (Agilent Technologies Italia S.p.A., Milan, Italy).

### 2.2. Adsorption of Nitrites with R1, R2, R1HG, and R2HG

To evaluate the efficiency of R1, R2, and the related hydrogels (R1HG and R2HG), aqueous samples artificially contaminated with nitrites were prepared by dissolving sodium nitrite (NaNO_2_) in water. Concentrations of nitrite in the untreated initial nitrite solutions and in the nitrite water solutions during and/or after treatment with adsorbents were measured by UV–Vis analyses using GRS and a calibration curve that was constructed as described in the following section.

#### 2.2.1. Calibration Curves

A stock solution (SS) of NO_2_^−^ 1000 mg/L (21.74 mM) was prepared by dissolving precisely 1.50 g of NaNO_2_ in 1000 mL deionized water. Then, by diluting 1 mL of the prepared SS to 100 mL with deionized water, a working solution (WS) at a concentration of 10 mg/L (0.22 mM) was obtained.

Optimal dilutions of the WS as reported in Table 1 were performed to obtain six 10 mL standard solutions (STD1–STD6). A 10 mL solution not containing nitrites was used as a blank.

Once the STD solutions and the blank were obtained, the GRS was added to each solution, according to Table 1, and about 20 min passed until the appearance of a pink/purple coloration.

After the colour changed, the absorbance (Abs) for all prepared solutions was measured using a UV–Vis spectrophotometer at 𝜆 max = 520 nm. Measures were performed in quadruplicate, and Abs values were reported as mean ± standard deviation (S.D.). The obtained values of Abs were plotted against the concentrations (mM), and a linear calibration curve was obtained with Microsoft Excel software using the ordinary least squares (OLS) method, the Equation (1) for which is reported below.

(1) y=33.419x

Equation (1) was used for determining the concentrations of the NO_2_^−^ ion in the appositely prepared contaminated water samples before and after treatment with the resins and hydrogels.

#### 2.2.2. Batch Experiments: Effect of Contact Time

Starting from the indication reported in a study on the removal of nitrate by different adsorbents [36], we prepared NO_2_^−^ solutions (100 mL) at the nominal concentration of 100 mg/L (2.17 mM), diluting 10 mL of the SS to 100 mL with deionized water. An aliquot of 100 µL was withdrawn before adding the adsorbents and optimally diluted to 10 mL to adapt it to the calibration curve. The obtained solution was treated with the GRS (250 µL), and about 20 min passed until the appearance of a purple coloration. The actual NO_2_^−^ concentrations at time 0 (T0) in the diluted solution (1.18 mg/L; 0.0257 mM) were measured spectrophotometrically at 𝜆 max = 520 nm. Then, according to literature data reporting on the use of adsorbents at concentrations in the range of 5–20 g/L [36], the residual undiluted solution (118 mg/L; 2.57 mM) was added with 0.5 g (5 g/L) of R1 or R2. The suspensions were kept under magnetic stirring, at pH = 7.5 and at room temperature (r.t.), until the amount of NO_2_^−^ ions adsorbed by R1 or R2 did not change further. The NO_2_^−^ concentrations in the suspensions were measured spectrophotometrically after 5, 15, 35, 65, 125, 215, and 335 min. In particular, aliquots were withdrawn at the fixed time points, and the residual adsorbents were separated by centrifugation (4000 rpm, 30 min). The obtained solutions were diluted at a ratio of 1:100 with deionized water, treated with the GRS, and their absorbance was measured at 𝜆 max = 520 nm. All the measurements were performed in quadruplicate, and the results of the absorbance were reported as means ± SD.

The removal efficiency of R1HG and R2HG formed in the suspensions was calculated from Equation (2):

(2) $$R\%=\frac{Co-Ct}{Co}\times 100$$

where *Ct* and *Co* are the concentration of NO_2_^−^ at any time and its initial concentration (mg/L or mM), respectively, and *R%* is the removal efficiency percentage.

The amounts of NO_2_^−^ adsorbed per unit mass of R1 and R2 (mg/g and mmol/g) at the fixed times (*Qt*) were determined according to Equation (3):

(3) $$Qt=\frac{\left[Co-Ct\right]\times V}{M}$$

where *Co* is the initial NO_2_^−^ concentration (mg/L or mM), *Ct* is the NO_2_^−^ concentration (mg/L or mM) at time *t*, *V* is the volume (L) of the NO_2_^−^ solution (0.1 L), and *M* is the mass of R1 or R2 (0.5 g). Finally, the amounts of NO_2_^−^ adsorbed at equilibrium (*Qe*) (mmol/g) by R1HG and R2HG were calculated from the mass balance Equation (4):

(4) $$Qe=\frac{\left[\left(Co-Ce\right)\times V\right]}{M}$$

where *Ce* and *Co* are the concentrations of NO_2_^−^ at equilibrium and its initial concentrations (mg/L or mM), respectively, *V* is the volume of the NO_2_^−^ solution (0.1 L), and *M* is the mass of R1 or R2 (0.5 g).

#### 2.2.3. Adsorption Experiments by Filtration

The experiments for adsorption by filtration were conducted using a plastic syringe as a column (with a volume capacity of 20 mL and a diameter of 1.5 cm) loaded with R1HG or R2HG as filtrating material. Specifically, the hydrogels were prepared by dispersing R1 (1 mL, 600 mg) or R2 (1 mL, 600 mg) in an excess of deionized water directly in the syringe. The resins were allowed to adsorb water under sonication (40 min), and once the resins reached their equilibrium degree of swelling (EDS) and their equilibrium water content (EWC), the excess of not adsorbed water was removed by applying compressed air from the top. A final volume of 10 mL of the hydrogels in the columns was reached. In the first experiment, a 100 mL solution containing NO_2_^−^ at a nominal concentration of 100 mg/L (2.17 mM) was prepared by diluting 10 mL of SS to 100 mL with deionized water. An aliquot of 100 µL was diluted to 10 mL to create a solution at the nominal concentration of NO_2_^−^ 1 mg/L (0.022 mM) and treated with the GRS (250 µL) until the appearance of a pink color. Then, its UV–Vis absorbance was measured at λ max 520 nm to obtain the actual concentration of the solution (1.18 mg/L; 0.0257 mM). Then, as reported [37], a volume of the original NO_2_^−^ solution (NO_2_^−^ concentration 118 mg/L; 2.57 mM) equal to the volume of the gels in the column (10 mL) was added from the top of the hydrogels and filtered through the gels under pressure until the complete emptying of the column. The filtrate was collected from the bottom of the column, diluted (1:100), treated with the GRS (250 µL) for 30 min, and analyzed by UV–Vis. The removal efficiency of R1HG and R2HG in the first experiment was calculated from Equation (5):

(5) $$R\%=\frac{Co-Cf}{Co}\times 100$$

where *Cf* and *Co* are the concentrations (mg/L or mM) of NO_2_^−^ in the filtrate and the initial solution before filtration, respectively.

In a subsequent experiment, without refreshing the hydrogels in the columns, an additional 50 mL of the NO_2_^−^ solution (118 mg/L; 2.57 mM) was filtered, and the effluent was collected every 5 mL. Ten samples were obtained, which were diluted at a ratio of 1:100, and the diluted solutions were separately treated with the GRS for 30 min (250 µL) and analyzed by UV–Vis (520 nm).

All measurements were performed in quadruplicate, and the results of absorbance were reported as means ± SD.

##### Regeneration of Adsorbents R1 and R2

The worn-out R1HG and R2HG from the previous experiments were recovered by filtration and regenerated by washing using a 1% HCl solution. The regenerated adsorbents were fully dried in an oven at 60 °C up to a constant weight before reuse.

## 3. Results and Discussion

### 3.1. Resins R1 and R2 and Related Hydrogels R1HG and R2HG

Recently, we reported on the high efficiency of two hydrogels (R1HG and R2HG) in removing anionic dyes from water by electrostatic binding. The gels were formed when polystyrene-based cationic resins (R1 and R2) that we prepared according to the Scheme S1 (see Section S1, Supplementary Materials) were dispersed in water [27]. Particularly, R1HG and R2HG demonstrated high removal efficiency both when used in batches (97–100%) and in columns (100%), mimicking possible decontamination systems working by contact or filtration [27]. In this regard, Table 2 summarizes some of the main physicochemical properties of R1, R2, R1HG, and R2 HG. Figures S2–S4 (see Section S2, Supplementary Materials) show the morphology and size of dried particles of R1 and R2 by optical microscopy and SEM, and Figure S5 shows the morphology and size of the swollen particles of R1HG and R2HG by optical microscopy. Table 3 summarizes the results concerning the removal of MO, F, and a mixture of the two (MOF) obtained by experiments in batches, and Figure S6 (Section S3, Supplementary Materials) shows the cumulative removal efficiency curves. Table 4 summarizes the results concerning the removal of MO, F, and MOF by filtration, obtained by experiments in a column. Figure S7 (Section S3 in Supplementary Materials) shows the UV–Vis spectra of the MO, F, and MOF solutions before filtration and after filtration through R1HG and R2HG. Finally, Table 5 collects the data from the kinetic studies, which established that in all cases, the removal of dyes by contact followed a pseudo-second-order (PSO) kinetic. Particularly, in Table 5, the values at the equilibrium adsorption state (*Qe*) calculated from the equations of the calibration kinetic models (*Qe* cal. (mg/g)) [27] and the constant values of the PSO kinetic models (*K2*) were reported. As can be seen, the values of *Qe* (cal.) (Table 5) were in reasonable agreement with the experimental ones (Table 3), thus establishing that the diffusion stage within the particle was not involved in the mechanism of the adsorption process [31].

### 3.2. Removal of Nitrites from Water by R1HG and R2HG

Nitrite was chosen as the representative compound of nitrogen pollution, since ion nitrite is an intermediate stage in the nitrogen cycle. Particularly, it is formed by the bacterial oxidation of ammonia, and by oxidation, it in turn provides the nitrate ion. The legal limit of nitrite in potable water is set at 0.5 mg/L, but usually, its concentration is below 0.1 mg/L. Therefore, is important to know the degree of pollution of the water samples [38].

Here, we tested the ability of the cationic hydrogels R1HG and R2HG, which self-formed when R1 and R2 were added to aqueous solutions, to adsorb nitrites by electrostatic interactions.

To study the capability of our materials to adsorb and retain nitrite ions, sodium nitrite was used to prepare water solutions artificially contaminated with nitrite at a NO_2_^−^ nominal concentration of 100 mg/L (2.17 mM). The removal efficiency of our adsorbents was first investigated by carrying out experiments in batches to assess their ability to capture and retain nitrite by contact over time. Secondly, we used columns filled with the adsorbents, mimicking mini decontamination-filtering systems to assess the capability of the adsorbents to remove nitrite by rapid filtration under pressure. In this case, the reduction in the removal efficiency of the gel columns during continuous filtration of the nitrite solutions was also investigated.

With the necessary dilutions, the concentration of nitrites in the prepared aqueous models of water contaminated with NO_2_^−^ ions before, during, and after treatment with the adsorbents was determined by UV–Vis spectrometry using the GRS method and Equation (1) of the linear calibration curve. The calibration curve was constructed with the OLS method, using STD solutions of NO_2_^−^ and measuring their absorbance with a UV–Vis spectrometer (520 nm) after their reaction with the GRS. The appearance of all standard solutions after the reaction with GRS is shown in Figure S8 (Supplementary Materials), and the obtained linear regression model and the related equation is shown in Figure S9 in the Supplementary Materials.

#### 3.2.1. The Griess Reagent System (GRS) Method

**Scheme 1:**
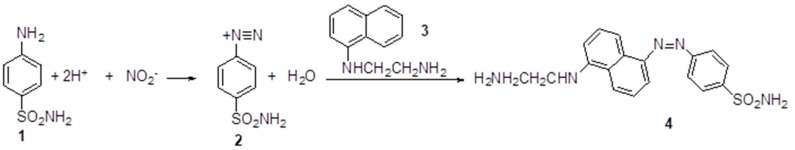
Reaction between nitrite ions and GRS providing the azo dye compound **4**.

So far, several techniques have been developed for nitrite detection. Among these, electrochemical and fluorescent methods have high sensitivity, but tedious sample preparation or instruments for signal transduction are required [39]. Nanometal–polymer composites (NMPCs), synthesized by the immobilization of inorganic metal quantum dots (especially noble transition metals) onto organic polymers, have demonstrated advanced optical and electrical properties, which are also exploitable for developing sensors and for detection purposes [40]. In particular, plasmonic nanoparticles have emerged as a promising tool for designing sensitive colorimetric detection schemes in recent years, due to their unique optical property of localized surface plasmon resonance (LSPR) dependent on their size, shape, and inter-particle distance [41,42,43]. Gold nanoparticles are among the most extensively studied plasmonic materials for colorimetric assay due to their strong LSPR and ease of chemical modification [41,42]. The Griess reagent system provides a simple and well-characterized colorimetric assay for the determination of nitrites with a detection limit of about 100 nM. The GRS measures the nitrite (NO_2_^−^) by relying on a diazotization reaction that was originally described by Griess in 1879 [44]. Through the years, many modifications to the original reaction have been described. Particularly, the GRS is based on a chemical reaction that can occur between sulfanilamide and N-1-naphthylethylenediamine dihydrochloride (NED) under acidic (phosphoric acid) conditions in the presence of NaNO_2_. At an acidic pH (pH 2.0–2.5), sodium nitrite affords nitrous acid, which nitrogenates the sulfanilamide, thus providing a diazonium salt that in turn couples with N-(1-naphthyl)-ethylenediamine, affording an azo dye compound. The obtained azo dye has a color that can vary from pink to red to purple based on its concentration, and it is possible to measure its absorbance at 520 nm. These reactions are detailed in Scheme 1.

This system detects NO_2_^−^ in a variety of biological and experimental liquid matrices such as plasma, serum, urine, and tissue culture medium. The nitrite sensitivity is dependent on the matrix. The limit of detection is 2.5 μM (125 pmol) nitrite (in ultrapure, deionized, distilled water) when the protocol described in Technical Bulletin #TB229 is used (available online at https://ita.promega.com/-/media/files/resources/protocols/technical-bulletins/0/griess-reagent-system-protocol.pdf?rev=8b478e337acd43be863d7787c80ea1c6&sc_lang=en, accessed on 11 March 2023).

#### 3.2.2. Batch Experiments: Removal of Nitrites from Water by R1HG and R2HG upon Contact

**Figure 1:**
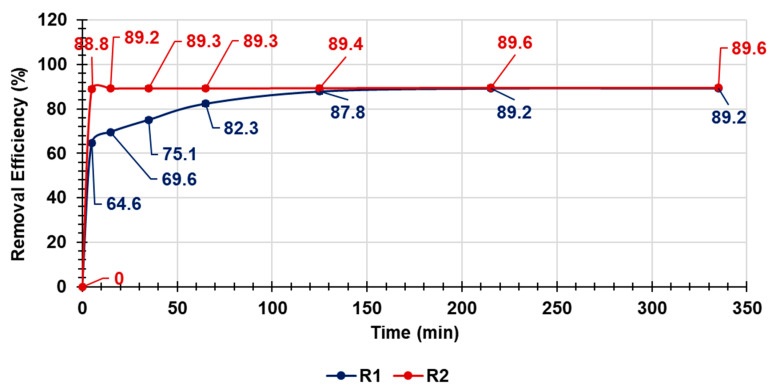
Cumulative removal efficiency (R%) (pH = 7.5; r.t.) of NO_2_^−^ by R1 (blue line) and R2 (red line). NO_2_^−^ initial concentration was 118 mg/L (2.57 mM). Error bars not detectable.

Typical experiments, as described in Section 2, were carried out according to recently reported indications [27,37]. Figures S10 and S11 (Section S4 in Supplementary Materials) show the appearance of NO_2_^−^ solutions treated with the GRS at time T0 (untreated solutions, cuvette 1 on the left), NO_2_^−^ solutions during treatment with R1 (Figure S10) and R2 (Figure S11) after 5, 15, 35, 65, 125, 215, and 335 min (cuvettes 2–8 from the left to right), and of blank solutions (cuvette B on the right). The UV–Vis spectra of solutions under treatment were measured at fixed time points (T5, T15, T35, T65, T125, T215, T335), after dilutions of 1:100 and treatment with GRS (250 µL). Using the absorbance data and the related concentrations of NO_2_^−^ obtained using Equation (1), we calculated the removal efficiency of both resins (*R%*), the amounts of NO_2_^−^ adsorbed per unit mass of R1 and R2 (mg/g and mmol/g) at the fixed times (*Qt*), and the amounts of NO_2_^−^ adsorbed by R1 and R2 at equilibrium (*Qe*) (mmol/g) using the Equations (1)–(3) (Section 2), respectively. The values of the removal efficiency (R%) of R1 and R2 were plotted vs. times, obtaining the cumulative removal percentage graphs reported in Figure 1.

As can be observed, both resins at equilibrium demonstrated a similar high removal efficiency (89.2% for R1; 89.6% for R2) in removing NO_2_^−^ ions from water, with R2 being more efficient and rapid than R1, as later confirmed by kinetic studies.

**Figure 2:**
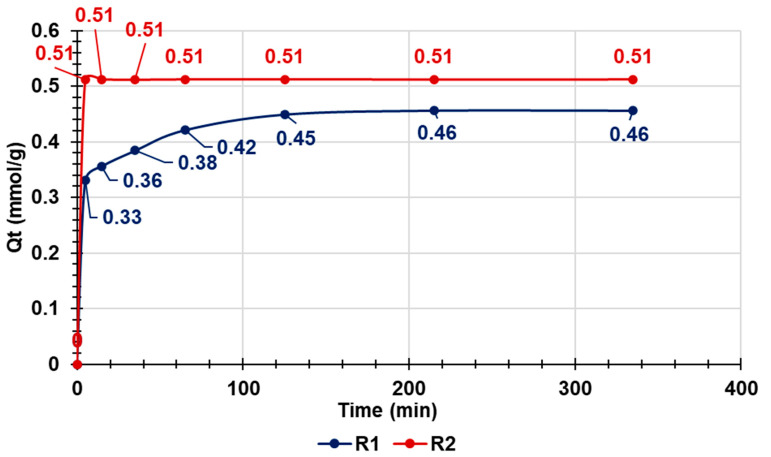
Time-dependent adsorption (mmol/g) of NO_2_^−^ on resins R1 (blue line) and R2 (red line) (pH = 7.5; r.t). NO_2_^−^ initial concentration was 118 mg/L (2.57 m). Error bars not detectable.

However, both resins reached their maximum removal efficiency after the same time of 215 min. Using Equation (3) reported in Section 2, we obtained the amounts of NO_2_^−^ adsorbed per unit mass of R1 and R2 (mmol/g) at the fixed times (*Qt*), which were plotted vs. times, obtaining the adsorption kinetic curves reported in Figure 2, whose trend was perfectly comparable to that of the cumulative removal efficiency in Figure 1.

As observable in Figure 2, the amount of NO_2_^−^ (mmol) removed per gram of adsorbent by resin R2 was 9.8% higher than that removed by R1. Additionally, R2 was significantly more rapid than R1, reaching the equilibrium adsorption status (*Qe*) remarkably earlier. In particular, R2 reached the *Qe* after 5 min, removing 0.51 ± 0.003 mmol/g (23.5 ± 0.14 mg/g) NO_2_^−^, whereas R1 succeeded in removing 0.46 ± 0.003 mmol/g (21.0 ± 0.14 mg/g) NO_2_^−^ after 215 min.

##### Kinetic Studies

To obtain important information on the pathways and mechanisms of the adsorption reactions, we carried out a kinetic study, as previously reported [27]. During the removal of nitrite using R1 and R2, kinetic models of pseudo-first-order (PFO) (Equation (6)), pseudo-second-order (PSO) (Equation (7)), and intra-particle diffusion (IPD) or Higuchi kinetics (Equation (8)) were studied [27,45]:

(6) $$ln\left(Qe-Qt\right)=lnQe-K1\times t$$

(7) $$\frac{t}{Qt}=\frac{1}{K2\times Qe^{2}}+\frac{1}{Qe}t$$

(8) $$Qt=Kint\times t^{0.5}+I$$

where *Qe* (mg/g) and *Qt* (mg/g) are the NO_2_^−^ adsorption capacities of R1 and R2 at equilibrium and at time *t,* respectively, *K*1 is the adsorption constant of the PFO kinetic model (1/min), *K*2 is the equilibrium constant velocity of the PSO kinetic model (g/m × gg), *K int* is the IPD rate constant (mg/g × min^0.5^), and *I* is the intercept of the linear curve (mg/g). The values of *ln*(*Qe* − *Qt*), *t/Qt,* and *Qt* were plotted vs. times, the times themselves, and the root square of the times (RST), respectively. The dispersion graphs obtained and their linear regression lines provided by Microsoft Excel software using the OLS method are available in the Supplementary Materials as Figures S12–S14. The coefficients of determination (R^2^) of the equations of the linear regressions obtained are reported in Table 6. The highest values of R^2^ were used as the parameters for determining the kinetic models that best fit the data of the adsorption processes and to decide which mechanisms governed the adsorption reactions.

The results showed that in both cases, the R^2^ values for the PSO models were higher than those obtained for other models. Consequently, the kinetic behavior of the adsorption of nitrite using both R1 and R2 as adsorbents followed the PSO model.

**Figure 3:**
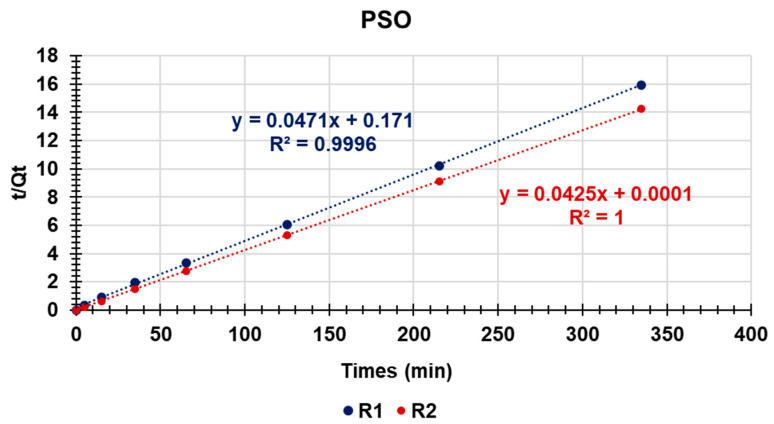
Pseudo-second-order kinetic models of nitrite removal using R1 (blue line) and R2 (red line). Qt expressed as mg/g; t/Qt = g/mg × min.

Figure 3 shows the PSO kinetic models of nitrite removal using R1 and R2.

In the processes governed by the PSO kinetics, the velocity-limiting step is considered to be chemical adsorption through the sharing or exchange of electrons between the adsorbent and the adsorbed or electrostatic interactions [27]. Therefore, as previously supposed, electrostatic interactions were confirmed to be the main mechanism by which both R1 and R2 adsorb nitrite.

The values of *Qe* (mg/g) estimated by the kinetic models (*Qe* (cal)) and *K2* were computed using the values of the slopes and intercepts of the equations in Figure 3 and are included in Table 7.

The values of *K2* established that R2 was able to remove nitrite remarkably faster than R1, and the calculated values of *Qe* perfectly agreed with the experimental ones, thus establishing that the diffusion stage within the particle was not involved in the mechanism of the adsorption process [31].

#### 3.2.3. Removal of Nitrite from Water upon Filtration on R1HG- and R2HG-Based Columns

Simulating a column system for the removal of nitrite from water by filtration, a plastic syringe loaded with R1HG or R2HG was used to perform a miniature-scale treatment of aqueous samples artificially contaminated with NO_2_^−^. In a typical procedure, R1 or R2 were inserted into the plastic syringe equipped with a small plastic-bottom cap [27]. The entire volume of the syringe was filled with water, and the resins were allowed to absorb water and swell at their EDS and EWC. The unabsorbed excess of water was removed from the bottom of the column apparatus by applying compressed air from the top. A typical column apparatus loaded with hydrogels is available in the Supplementary Materials of our recent work [27]. By controlling the initial volume of R1 and R2, we could easily control the final volume of hydrogels and, consequently, the length of the gel-columns.

**Figure 4:**
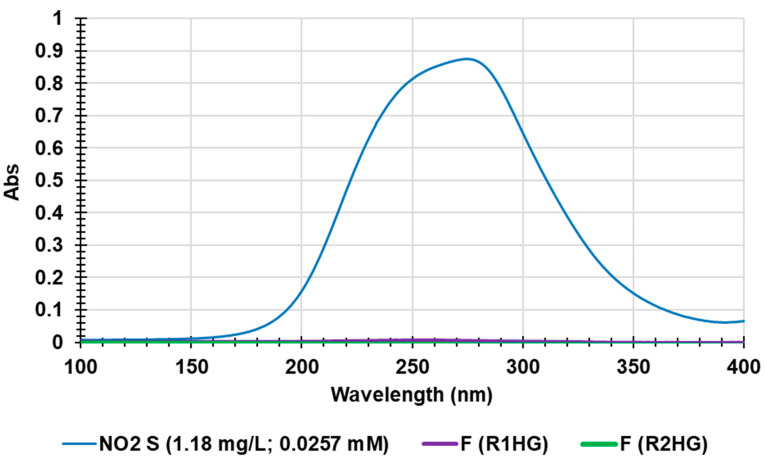
UV–Vis spectra of NO_2_^−^ solutions after treatment with GRS before filtration (blue line) and after filtration through R1HG (purple line) and R2HG (green lines). Columns: 10 mL hydrogels; pH = 7.5; room temperature (r.t.). Filtrations were carried out by applying compressed air to the top of the columns to completely empty the columns. NO2 S = NO_2_^−^ solution (118 mg/L; 2.57 mM) after dilution 1:100; F (R1HG) and F (R2HG) = NO_2_^−^ solutions (118 mg/L; 2.57 mM) after filtration by R1HG and R2HG, respectively, and after dilution of 1:100.

To assess the efficiency of the gel columns in adsorbing NO_2_^−^ by electrostatic interactions with ammonium groups, two types of sequential experiments were carried out. First, R1-based and R2-based gel columns loaded with 10 mL hydrogels were utilized to assess their efficiency (%) in removing nitrite from a nitrite solution of 118 mg/L with a volume equal to the volume of gel (10 mL) into the columns. As described in Section 2, an optimally diluted NO_2_^−^ solution was treated with the GRS until the appearance of a pink coloration (Figure S15, Supplementary Materials) to assess its actual concentration of NO_2_^−^ ions (1.18 mg/L; 2.57 mM) by UV–Vis at 520 nm. Then, 10 mL of the undiluted NO_2_^−^ solution was filtered through the gel columns under pressure until their complete emptying. After reaction with the GRS, no pink coloration was observed using either R1 or R2 (Figure S15, Supplementary Materials), thus evidencing the capability of the gels to retain NO_2_^−^ ions. The actual concentration of NO_2_^−^ ions in the filtrates was determined by UV–Vis analyses. Figure 4 shows the UV–Vis spectra of the NO_2_^−^ solutions treated with GRS before and after filtration through both R1HG and R2HG. As observable in Figure 4, the characteristic absorption peak of the dye obtained by reacting NO_2_^−^ in a solution with the GRS disappeared in the UV–vis spectra of the water solutions filtered through both R1HG and R2HG. Particularly, the determined concentrations of NO_2_^−^ in the solutions before and after filtration by R1HG and R2HG and the removal efficiency percentages of the two resins are reported in the first row (V = 10 mL) of Table 8 and Table 9, respectively.

**Figure 5:**
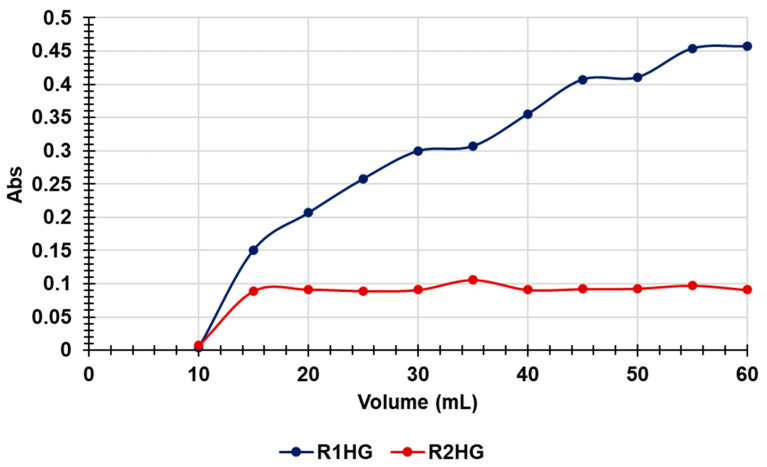
Abs values as a function of the volumes of NO_2_^−^ solutions 118 mg/L (2.57 mM) sequentially filtered under pressure through R1HG (blue line) and R2HG (red lines). Columns: 10 mL hydrogels; pH = 7.5; room temperature (r.t.). Error bars not detectable.

According to the data in Table 8 and Table 9, both R1HG and R2HG demonstrated high removal efficiency (99.53% for R1HG and 100.00% for R2HG) upon rapid filtration under pressure and a minimum contact of 10 mL NO_2_^−^ solutions 118 mg/L (2.57 mM). Particularly, they were capable of totally purifying a volume of contaminated water equal to that of the gels in the column and 10 times that of the R1 and R2 used. As observed in the experiments in batches, as well as in our previous recent study reporting on the capacity of R1HG and R2HG in removing anionic dyes from water [27], R2HG proved to be more efficient than R1HG. Once the capability of both resins in adsorbing NO_2_^−^ ions by rapid filtration of a 10 mL nitrite solution was established, we studied the reduction in the removal efficiency of the gel columns as a function of the volumes filtered, during the continuous filtration of increasing volumes of the nitrite solutions. Particularly, without refreshing the gels in the columns, after filtration of 10 mL NO_2_^−^ solutions, additional aliquots (5 mL) of the NO_2_^−^ solutions (118/mg/L; 2.57 mM) were sequentially filtered, diluted 0.1/10, treated with the GRS, and analyzed by UV–Vis. The experiments were interrupted after collecting 10 filtrates (total volume = 60 mL). Then, by plotting the values of Abs measured vs. those of volumes of NO_2_^−^ solutions that were filtered, we obtained the graphs in Figure 5.

**Figure 6:**
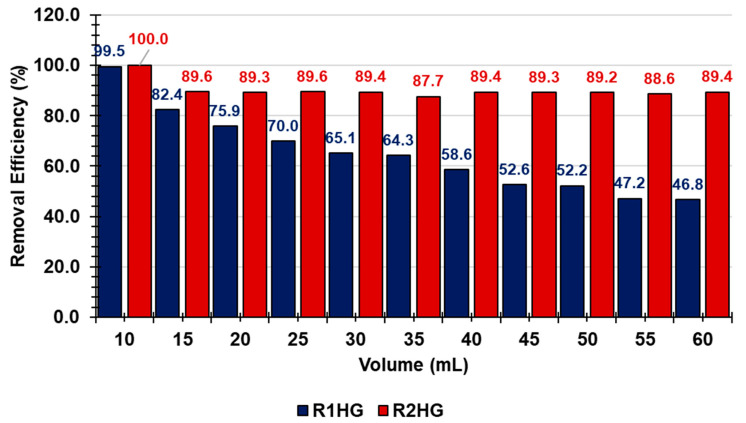
Removal efficiency values of R1HG and R2HG during filtration of increasing volumes of NO_2_^−^ solutions 118 mg/L (2.57 mM).

If R1HG was only slightly less efficient than R2HG in removing the NO_2_^−^ ion from water in the filtration of the first 10 mL of nitrite solutions, here, it performed considerably less well than R2HG. In fact, after having filtered 10 mL of NO_2_^−^ solution, the Abs values of R2HG slightly increased only upon the filtration of a further 5 mL and then remained constant. Under 0.1 up to the filtration of 60 mL (NO_2_^−^ concentration in the filtrate 0.2696 mM, R = 89.42%), the values of R1HG increased steadily to reach Abs = 0.4571 after the filtration of 60 mL (NO_2_^−^ concentration in the filtrate 1.36 mM, R = 46.8%). These results established that although R2HG maintained a saturation of about 10% after having also filtered 60 mL of NO_2_^−^ 118 mg/L, thus allowing a constant removal efficiency of >89% (Figure 6), the saturation of R1HG increased steadily, thus causing a rapid decline in its removal efficiency down to <50% upon the filtration of 55 mL (Figure 6).

Accordingly, R2HG was able to efficiently filter (R > 89%) a volume of contaminated water six-fold the volume of gel in the column and sixty times the volume of R2 used. As reported previously [27], the higher efficiency of R2HG is probably due to the presence of an additional methylene group in the carbon linker between the phenyl ring and the -NH_3_^+^ group in the structure of R2. Being more distant from the ring, the cationic group is freer to electrostatically interact with the negative charges of the NO_2_^−^ ion, thus increasing the adsorption efficiency of R2HG compared to R1HG. Additionally, R2HG could remove a higher amount of NO_2_^−^ than R1HG without losing efficiency because of its higher content of cationic -NH_3_^+^ groups. These results demonstrated that the as-fabricated gel-columns possess a strong capability to remove anionic ion NO_2_^−^ from water on a miniature scale, and those made with R2 might be especially promising candidates to be applied in the treatment of drinking water that is highly contaminated with nitrites. According to a recent report (Nitrites/Nitrates in Water: What You Need to Know in 2023, by Jennifer Byrd Jennifer Byrd available online at https://waterfilterguru.com/nitrites-nitrates-in-water/ (accessed on 26 March 2023)), the maximum contaminant level of nitrite-nitrogen in drinking water derived from livestock and human sewage, industrial waste, and leaking septic tanks detected in 2023 was 1.0 mg/L. In this regard, both of our adsorbents could be capable of reducing the nitrite concentration under the legal limit of 0.5 mg/L through simple filtration, from volumes of such contaminated water 50–60-fold higher than the volumes of resins used. Particularly, R1 would be capable of decreasing the nitrite concentration at 0.48 mg/L, filtering a volume of water fifty times higher than the volume of resin used. Meanwhile, R2 would be capable of decreasing the nitrite concentration at 0.11 mg/L, filtering a volume of water sixty times higher than the volume of resin used.

##### Saturation Kinetics Studies

We investigated the mechanisms of saturation and of the reduction in the removal efficiency of R1HG and R2HG during the filtration process carrying out a kinetic study, like that conducted for adsorption reactions (Section 3.2.2). To this end, the PFO, PSO, and IPD kinetic models were applied to the data of graphs in Figure S16 (Supplementary Materials). The graphs were obtained by plotting the NO_2_^−^ concentrations revealed in the effluents during filtration vs. the increasing volumes filtered in the range of 10–60 mL (Section 3.2.2).

In this case, the PFO, PSO, and IPD kinetic models are expressed by Equations (9)–(11):

(9) $$ln\left(Q60-QV\right)=lnQ60-K1\times V$$

(10) $$\frac{V}{QV}=\frac{1}{K2\times Q60^{2}}+\frac{1}{Q60}V$$

(11) $$QV=Kint\times V^{0.5}+I$$

*Q60* (mM) and *QV* (mM) are the NO_2_^−^ concentration in the effluents at the bottom of the R1HG and R2HG columns after the filtration of 60 mL and after the filtration of a volume V, respectively. *K*1 is the kinetic constant of the PFO kinetic model (1/mL), *K*2 is the equilibrium constant velocity of the PSO kinetic model (g/mM), *K int* is the IPD rate constant (mM × mL^0.5^), and *I* is the intercept of the linear curve (mM). The values of *ln*(*Q*60 − *QV*), *V/QV,* and *QV* were plotted vs. volumes, the volumes themselves, and the root square of the volumes (RSV), respectively. Table 10 reports the coefficients of determination (R^2^) of all the equations of the linear regressions obtained for all kinetic models.

The results showed that in the case of R1HG, a higher value of R^2^ was obtained for the IPD model, whereas in the case of R2HG, a higher value was obtained for the PSO model. Consequently, the kinetic of saturation and of the reduction in the removal efficiency of R1HG and R2HG used as adsorbents during protracted filtrations followed the IPD and PSO models, respectively.

**Figure 7:**
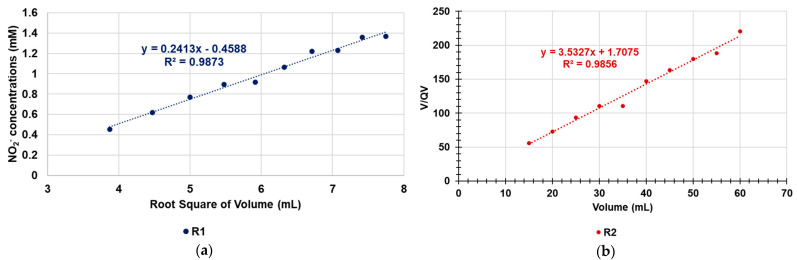
IPD kinetic model obtained for R1HG (**a**) and PSO kinetic model obtained for R2HG (**b**). QV was expressed as mM; V/QV = mL/mM.

Figure 7 shows the IPD and PSO kinetic models obtained for R1HG (Figure 7a) and for R2HG (Figure 7b).

The values of *K int, I, Q60* (cal), and *K2* were estimated with the equations of the kinetic models in Figure 7a,b and are included in Table 11.

These results established that although the saturation of R1HG and, consequently, the increase in nitrite concentration in effluents was mainly governed by diffusion (IPD kinetic model), that of R2HG involved chemical processes, such as the sharing or exchange of electrons between the adsorbent and the adsorbed or electrostatic interactions [27]. The maximum value of the concentration of nitrites not absorbed and still present in the filtered water (*Q60*) calculated for R2HG with the equation of the PSO kinetic model (*Q60* cal = 0.28 mM) was in perfect agreement with experimental data (*Q60* = 0.2720 mM), thus excluding diffusion mechanisms [31].

##### Regeneration of Adsorbents R1 and R2

The possibility to regenerate and reuse adsorbents, without significant changes in the adsorption capacity and efficiency, is pivotal in reducing the cost of the adsorption process and synthesized adsorbent. To test the reusability and regeneration capability of our materials, R1HG and R2HG from the previous experiments were recovered by filtration and were washed and regenerated using a 1% HCl solution, as previously reported [46]. The regenerated adsorbents (R1HG-R and R2HG-R) were fully dried in an oven at 60 °C up to a constant weight before each reuse.

**Figure 8:**
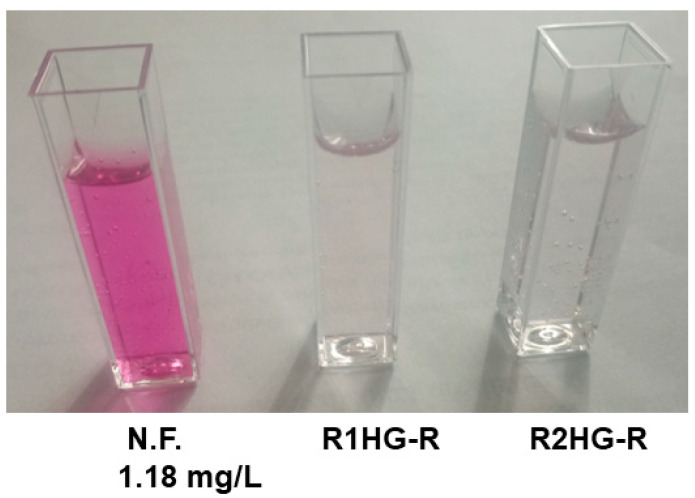
Nitrate solution before filtration (N.F.) and filtrates by regenerated R1HG-R and R2HG-R by washing with 1% HCL.

Then, R1HG-R- and R2HG-based columns like those described in Section 3.2.3 were prepared and used to filter 10 mL of the nitrite solutions of 118 mg/L (2.57 mM), as previously described. As shown in Figure 8, neither filtrate assumed a pink coloration after dilution at 1:100 and treatment with the GRS, which was different from the unfiltered (N.F.) solution treated in the same conditions.

**Figure 9:**
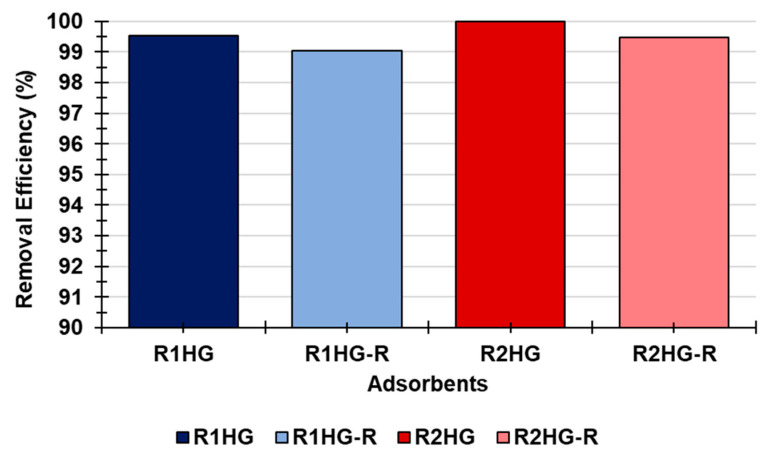
Removal efficiency of R1HG and R2HG compared with that of regenerated R1HG-R and R2HG-R.

The filtrates were finally analyzed spectroscopically by UV–Vis to assess the removal efficiency of the regenerated resins. Table 12 reports the results, and Figure 9 compares the removal efficiency of R1HG and R2HG with that of R1HG-R and R2HG-R.

The removal efficiency was reduced in both cases by 0.5%, thus establishing that upon regeneration, the removal efficiency of both resins underwent only minimal changes. In order to confirm that the removal efficiency of the regenerated gels was not significantly different from that of R1HG and R2HG, a student’s *t* test was carried out considering both 90% and 99% confidence intervals. From the results, it was established that no significant statistical difference existed between R1HG and R1HG-R, nor between R2HG and R2HG-R, at a 90% confidence interval, and no significant statistical difference existed between all samples at a 99% confidence interval, thus evidencing that no morphological robustness, chemical compositional, or oxidation state changes occurred during the process of adsorption, thus confirming the stability of both our absorbents.

## 4. Conclusions

Although levels over 0.5–1 mg/L of total nitrogen in water have dramatic consequences for both humans and the aquatic ecosystem, and nitrite is known to be toxic to almost all living organisms, the literature is lacking in studies reporting on new materials and methods to remove nitrite from water. For the first time, in this work, we proposed low-cost, efficient solutions for the treatment of drinking water contaminated by nitrites. To this end, we evaluated the efficiency of two polystyrene-based cationic resins (R1 and R2) that provided hydrogels (R1HG and R2HG) with high EDS and EWC by simple dispersion in water to adsorb and retain NO_2_^−^ ions.

In adsorption experiments carried out in different conditions and monitored with UV–Vis methods using the Griess reagent system to detect the nitrite concentrations, R1HG and R2HG showed high adsorption efficiency based on electrostatic interactions, both by contact (>98%) and by filtration (99.5–100%). The results from the kinetic studies established that the kinetic behavior of the adsorption of nitrites using both R1 and R2 as adsorbents followed the pseudo-second-order kinetic model. In this regard, the values of K2 established that R2 was able to remove nitrites faster than R1, and, since the values of *Qe* estimated by the kinetic linear equations perfectly agreed with experimental ones, it was established that diffusion was not involved in the adsorption removal of nitrites from water. During filtration experiments, the removal efficiency of R1HG was very high (99.5%) upon the filtration of a volume of nitrite solution of 118 mg/L or 10 times the volumes of R1 used. However, it decreased to 82% and down to 47% for filtration of further volumes. Conversely, R2HG maintained a constant removal efficiency >98%, also upon the filtration of a volume 60 times higher than the volume of R2 used. Interestingly, both the worn-out hydrogels were completely regenerable by 1% HCl washing, without a significant reduction in their original efficiency (0.5%), thus confirming their stability during usage. Collectively, R1HG and especially R2HG proved to be low-cost, up-scalable, and regenerable adsorbent materials that are promising for rapid nitrite remediation and drinking water decontamination by simple contact or filtration. Specifically, R1HG and R2HG might be able to reduce the nitrite concentration under the legal limit of 0.5 mg/L (0.48 mg/L for R1HG and 0.11 mg/L for R2HG) in volumes of drinking water 50–60 times higher than the volumes of resin used, having the maximum contamination level registered in 2023, without resorting to more expensive and complicated currently adopted methods. Since the literature is lacking in papers concerning the development of new methods to remove nitrites from water, the present study is of immense relevance and novelty. We hope that the present work might be a pioneer for more extensive literature on the subject of nitrite remediation and inspire other scientists to perform research in the field.

## Figures and Tables

**Table 1:** Dilutions of WS and final concentrations of the STD1–6 solutions.

Solution	WS Aliquot (mL)	Water Added (mL)	Standard Concentrations (mg/L; mM)	GRS (µL)
STD1	0.2	9.8	0.2; 0.0043	250
STD2	0.3	9.7	0.3; 0.0065	250
STD3	0.5	9.5	0.5; 0.0109	250
STD4	0.75	9.25	0.75; 0.0163	250
STD5	1	9	1; 0.0217	250
STD6	2	8	2; 0.0435	500
Blank	0	10	0; 0	250

**Table 2:** Main physicochemical properties of R1, R2, R1HG, and R2HG.

Entry	Size	−NH_3_^+^	R1/R2 Concentrations	EDS ^1^	EWC ^2^	Weight Loss	ESR ^3^
	(µm)	(mmol/g)	(mg */mL)(% wt */v)	(%)	(%)	(%)/min	(%)/min
**R1**	125–250	13.20 ± 0.062	N.A.	N.A.	N.A.	N.A.	(1226)/10
**R2**		16.15 ± 0.062					(1669)/20
**R1HG**	500–550	0.9972 ± 0.0057	82.7 (8.3)	900	90.0	(94)/410	N.A.
**R2HG**		1.0373 ± 0.0059	68.5 (6.9)	1000	90.9	(94)/340	

* Weight of R1 and R2; ^1^ equilibrium degree of swelling; ^2^ equilibrium water content; ^3^ equilibrium swelling rate; N.A. = not applicable.

**Table 3:** Values of *Qe* and related times necessary to reach it.

	F	MO	F in MOF	MO in MOF
*Qe* (mg/g)	47.8 (R1); 49.0 (R2)	47.7 (R1); 48.5 (R2)	65.3 (R1); 65.2 (R2)	40.3 (R1); 40.3 (R2)
Time *	90 (R1); 20 (R2)	120 (R1); 30 (R2)	90 (R1); 60 (R2)	90 (R1); 30 (R2)

* Minutes.

**Table 4:** Results from adsorption experiments in column upon filtration.

Entry	MO	F	MOF	
	Abs; mg/L *	Abs; mg/L *	Abs; mg/L *	
Not treated	0.3488 ± 0.0001; 44.3 ± 0.1	0.9700 ± 0.0002; 50.3 ± 0.9	0.4187 ± 0.0152; 107.0 ± 0.14 ^2^	0.6894 ± 0.0037; 71.0 ± 0.14 ^3^
R1HG	0.0058 ± 0.0013; 0 **	0.0039 ± 0.0007; 0 **	−0.0044 ± 0.0001; 0 ^2^	−0.0031 ± 0.0002; 0 ^3^
R1HG R (%) ^1^	100	100	100	100
R2HG	0.0079 ± 0.0009; 0 **	0.0047 ± 0.0014; 0 **	−0.0051 ± 0.0002; 0 ^2^	−0.0038 ± 0.0001; 0 ^3^
R2HG R (%) ^1^	100	100	100	100

* Before dilutions; ** concentration of dye measured after filtration of both refreshed hydrogels before filtration of the second dye (F) and without refreshing them; ^1^ removal efficiency; ^2^ referred to MO in MOF; ^3^ referred to F in MOF.

**Table 5:** Values of *Qe* (cal) and *K*2.

	F	MO	F in MOF	MO in MOF
*Qe* (cal) *(mg/g)	47.8 (R1); 49.0 (R2)	47.8 (R1); 47.8 (R2)	64.5 (R1); 65.4 (R2)	40.3 (R1); 40.7 (R2)
*K*2 **	8.4 (R1); 34 (R2)	1.6 (R1); 5.5 (R2)	6.2 (R1); 38.2 (R2)	5.1 (R1); 15.4 (R2)

* Computed from the slopes of the equations of calibration models; ** computed from the intercepts of equations of calibration models [27].

**Table 6:** Values of coefficients of determinations (R^2^) obtained for all kinetic models considered.

Kinetic Model	R1	R2
PFO	0.4374	0.1189
PSO	0.9996	1
IPD	0.5416	0.2641

**Table 7:** Values of *Qe* (cal) and *K*2.

	R1	R2
*Qe* (cal) *(mg/g)	21.2 (21.0) ^#^	23.5 (23.5) ^#^
*K*2 **	0.0130	18.1

* Computed from the slopes of the equations in Figure 3; ** computed from the intercepts of equations in Figure 3; ^#^ experimental values of *Qe* (mg/g).

**Table 8:** Results from adsorption experiments in column upon filtration.

Volume mL	Not Treated Abs (mM) *	R1HG Abs (mM) **	R2HG Abs (mM) **
10	0.8559 ± 0.0055 (2.57 ± 0.02)	0.0040 ± 0.0002 (0.0120 ±0.0006)	−0.0071 ± 0.0002 (−0.0212 ± 0.0006)
15		0.1511 ± 0.0002 (0.4521 ± 0.0006)	0.0894 ± 0.0001 (0.2675 ± 0.0003)
20		0.2066 ± 0.0002 (0.6182 ± 0.0006)	0.0915 ± 0.0003 (0.2738 ± 0.0009)
25		0.2578 ± 0.0003 (0.7714 ± 0.0009)	0.0894 ± 0.0003 (0.2675 ± 0.0009)
30		0.2996 ± 0.0007 (0.8964 ± 0.0021)	0.0909 ± 0 (0.2720 ± 0)
35		0.3069 ± 0.0004 (0.9183 ± 0.0021)	0.106 ± 0.0002 (0.3172 ± 0.0006)
40		0.3552 ± 0.0004 (1.0629 ± 0.0021)	0.0911 ± 0.0004 (0.2726 ± 0.0012)
45		0.4072 ± 0.0004 (1.2185 ± 0.0021)	0.0922 ± 0.0004 (0.2759 ± 0.0012)
50		0.4105 ± 0.0003 (1.2283 ± 0.0009)	0.0929 ± 0.0002 (0.2780 ± 0.0006)
55		0.4538 ± 0.0004 (1.3579 ± 0.0021)	0.0976 ± 0.0024 (0.2920 ± 0.0072)
60		0.4571 ± 0.0002 (1.3678 ± 0.0006)	0.0909 ± 0.0002 (0.2720 ± 0.0006)

* NO_2_^−^ concentration (mM) in the nitrite solution before dilution and before filtration; ** NO_2_^−^ concentration (mM) in the nitrite solution before dilution and after filtration.

**Table 9:** Values of removal efficiency (%) after the filtration of 10–60 mL of NO_2_^−^ solutions 118 mg/L; 2.57 mM.

Volume (mL)	R1HG R (%)	R2HG R (%)
10	99.53	100.00
15	82.41	89.59
20	75.95	89.35
25	69.98	89.59
30	65.12	89.42
35	64.27	87.66
40	58.64	89.39
45	52.59	89.26
50	52.20	89.18
55	47.16	88.64
60	46.78	89.42

**Table 10:** Values of coefficients of determinations (R^2^) obtained for all kinetic models considered.

Kinetic Model	R1HG	R2HG
PFO	0.7707	0.4111
PSO	0.8784	**0.9856**
IPD	**0.9873**	0.3609

**Table 11:** Values of *K int, I*, *Q60* (cal), and *K*2.

	R1HG	R2HG
*K int*	0.2413	N.A.
*I*	0.4588	N.A.
*Q60* (cal) (mM)	N.A.	0.2831
*K*2	N.A.	7.3115

N.A. = not applicable.

**Table 12:** Results from adsorption experiments in column upon filtration by regenerated hydrogels (R1HG-R and R2HG-R).

Not Treated Abs (mM) *	R1HG-R Abs (mM) ** R (%)	R2HG-R Abs (mM) ** R (%)
0.8559 ± 0.0055 (2.57 ± 0.02)	0.0082 ± 0.0002 (0.02453 ± 0.0006) 99.04 (99.53) ***	0.0045 ± 0.0002 (0.0135 ± 0.0006) 99.48 (100.00) ***

* NO_2_^−^ concentration (mM) in the nitrite solution before dilution and before filtration; ** NO_2_^−^ concentration (mM) in the nitrite solution before dilution and after filtration; *** removal efficiency of freshly prepared hydrogels.

## Data Availability

All data concerning this work are included in the present manuscript and in the Supplementary Materials associated with this paper.

## Supplementary Materials

The following supporting information can be downloaded at: https://www.mdpi.com/article/10.3390/toxics11040312/s1, Figure S1. Chemical structure of monomers M1 and M2. Scheme S1. Reverse suspension co-polymerization of M1 and M2 to achieve R1 and R2, respectively. Numbers 1 and 2 indicate the number of carbon atoms of the chain linking the styrene ring and the ammonium group in M1, M2, as well as in R1, R2. Figure S2. Optic images (using a 4 × objective) of R1 showing microdimensioned particle of 178 µm (a) and 217 µm (b). Figure S3. SEM image of resin R1. Figure S4. Optic images (using a 4 × objective) of R2 showing micro-dimensioned particles of 171 µm (a) and 166 µm (b); SEM images of R2 (c,d). Figure S5. Optical microphotographs (using a 4 × objective) of R1HG (a,b) and R2HG (c,d), at their EDS. Figure S6. Cumulative removal efficiency (R%) (pH = 7.5; r.t.) of MO by R1 (red line) and R2 (blue line) (a); cumulative removal efficiency (R%) (pH = 7.5; r.t) of F by R1 (purple line) and R2 (green line) (b); cumulative removal efficiency (R%) (pH = 7.5; r.t) of MO (red line) + F (green line) (MOF) by R1 (c) and by R2 (d). F initial concentrations were 48.7 mg/L (R1), 49.4 mg/L (R2), 64.6 mg/L in MOF solution treated with R1, 64.9 mg/L in MOF solution treated with R2. MO initial concentrations were 50.9 mg/L (R1), 50.8 mg/L (R2), 39.6 mg/L in MOF solution treated with R1, 39.7 mg/L in MOF solution treated with R2. Figure S7. UV-Vis spectra of MO (a,c), F (b,d) and MOF (e) solutions before filtration (red or green lines) and after filtration through R1HG (purple lines) and R2HG (light blue lines). Figure S8. NO_2_^−^ standard solutions (STD1-SDT6) in the concentration range (0.2–2 mg/L; 0.0043–0.0435 mM) and blank (B) reacted with Griess reagent system (GRS) and used to construct the calibration curve shown in Figure S9. Figure S9. NO_2_^−^ calibration curve in the concentration range 0.0043–0.0435 mM (0.2–2 mg/L). Figure S10. Appearance of NO_2_^−^ solutions treated with the GRS at time T0 (untreated solution, cuvette 1 on the left), of NO_2_^−^ solutions during treatment with R1 after 5, 15, 35, 65, 125, 215 and 335 min (cuvette 2–8 from the left to right) and of the blank solution (cuvette B). Figure S11. Appearance of NO_2_^−^ solutions treated with the GRS at time T0 (untreated solution, cuvette 1 on the left), of NO_2_^−^ solutions during treatment with R2 after 5, 15, 35, 65, 125, 215 and 335 min (cuvette 2–8 from the left to right) and of the blank solution (cuvette B). Figure S12. PFO kinetic model (Qe and Qt expressed as mg/g). Figure S13. PSO kinetic model (Qt expressed as mg/g; t/Qt = g/mg × min). Figure S14. IPD kinetic model (Qt expressed as mg/g). RST = root square of times. Figure S15. NO_2_^−^ solutions before filtration (N.F.) and after filtration by R1 (from the left second cuvette) and by R2 (from the left third cuvette). Figure S16. Nitrite concentration in the solutions filtered by R1HG (blue line) and R2HG (red line) as a function of the volumes of NO_2_^−^ solutions 118 mg/L (2.57 mM) sequentially filtered under pressure. Columns: 10 mL hydrogels; pH = 7.5; room temperature (r.t.). Error bars not detectable.
