# Supplementary material for: Cationic Polystyrene-Based Hydrogels: Low-Cost and Regenerable Adsorbents to Electrostatically Remove Nitrites from Water
Source: Toxics. 2023 Mar 27;11(4):312. doi: 10.3390/toxics11040312 (PMC10142137; doi:10.3390/toxics11040312)
Supplement: Supplementary file 1 [file toxics-11-00312-s001.zip › toxics-2307106-supplementary.pdf]

Section S1. Chemistry

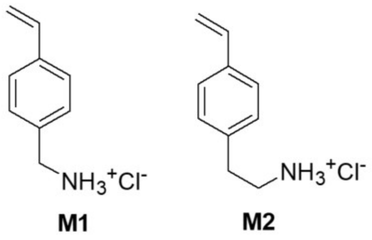

Figure S1. Chemical structure of monomers M1 and M2.

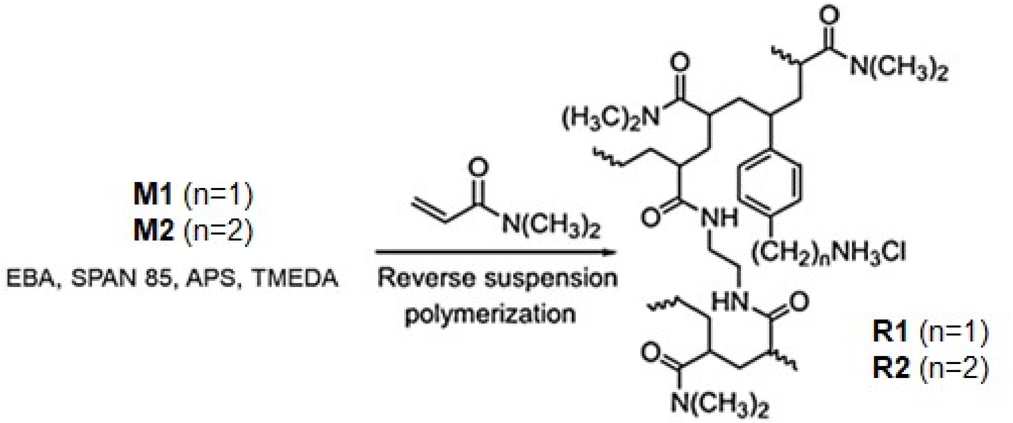

Scheme S1. Reverse suspension co-polymerization of M1 and M2 to achieve R1 and R2, respectively. Numbers 1 and 2 indicate the number of carbon atoms of the chain linking the styrene ring and the ammonium group in M1, M2, as wellas in R1, R2.

Sections S2. Analysis of the miscrostructure of R1 and R2.

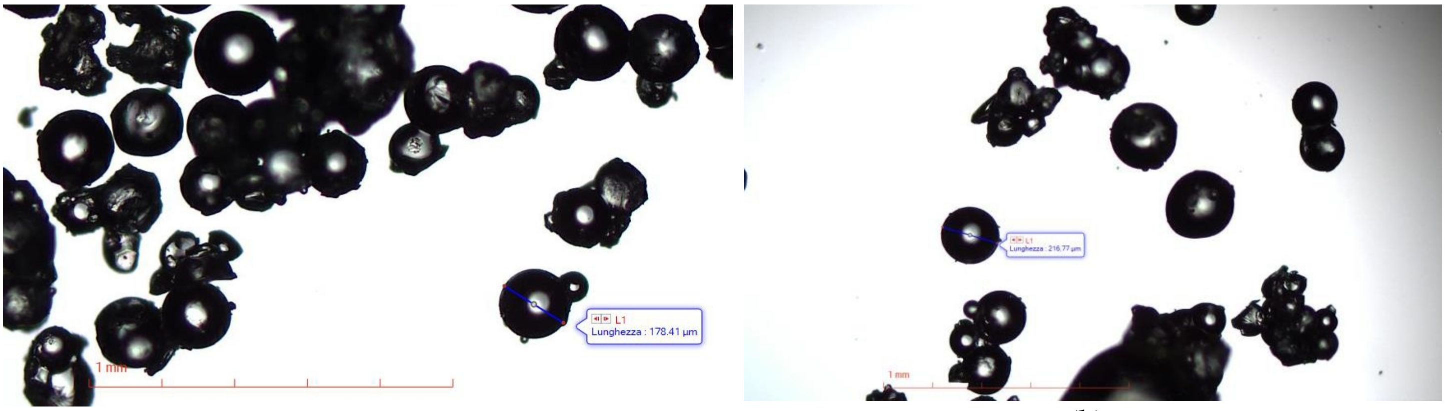

(**a**) (**b**)

Figure S2. Optic images (using a 4 × objective) of R1 showing microdimensioned particle of 178 µm (**a**) and217 µm (**b**).

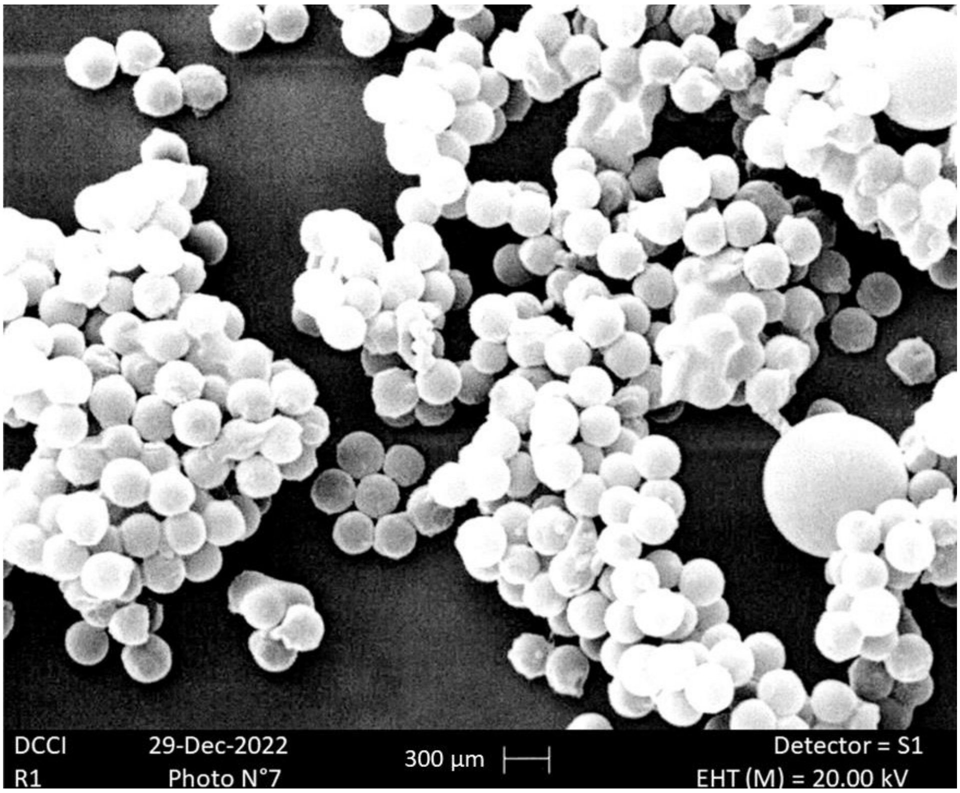

Figure S3. SEM image of resin R1.

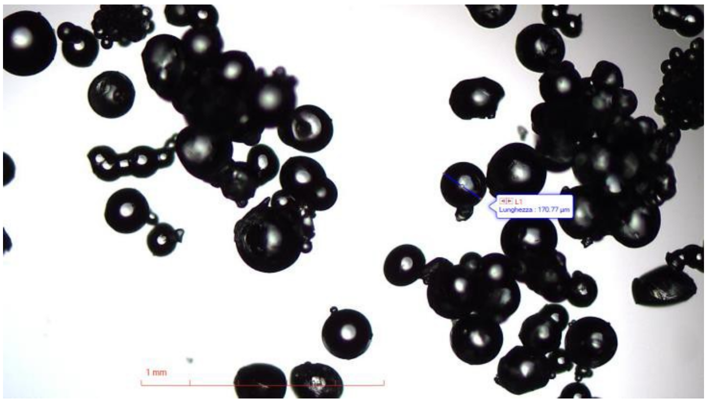

(**a**)

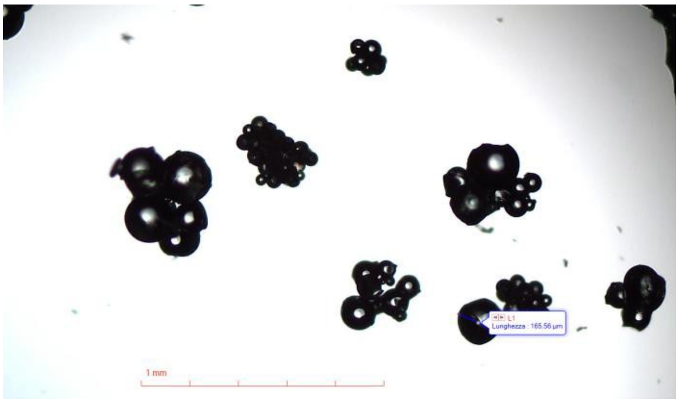

(**b**)

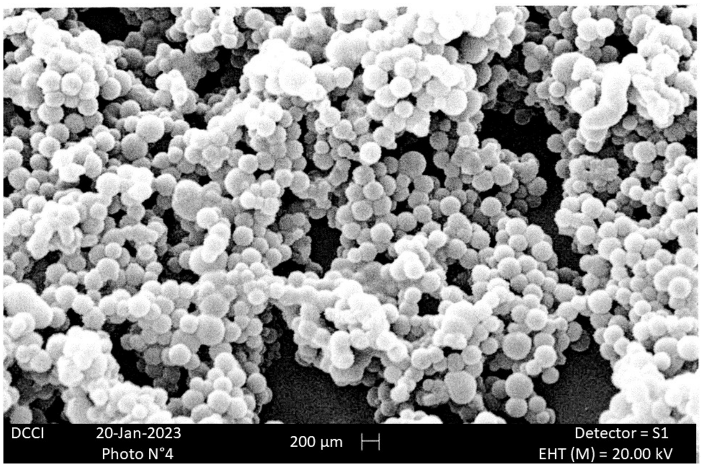

(**c**)

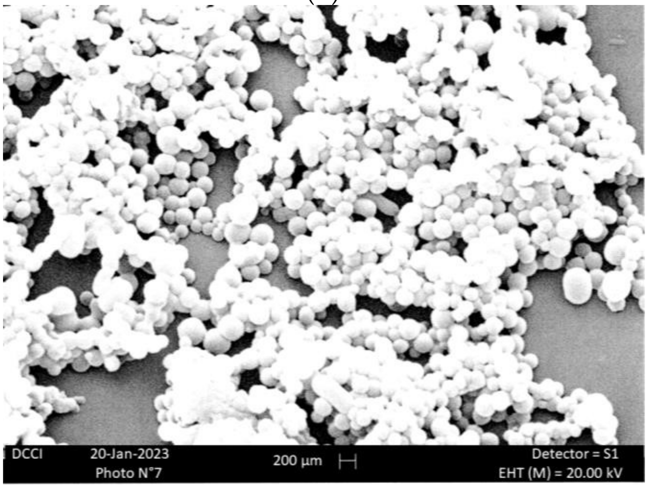

(**d**)

Figure S4. Optic images (using a 4 × objective) of R2 showing micro-dimensioned particles of 171 µm (**a**) and 166 µm (**b**); SEM images of R2 (**c**, **d**).

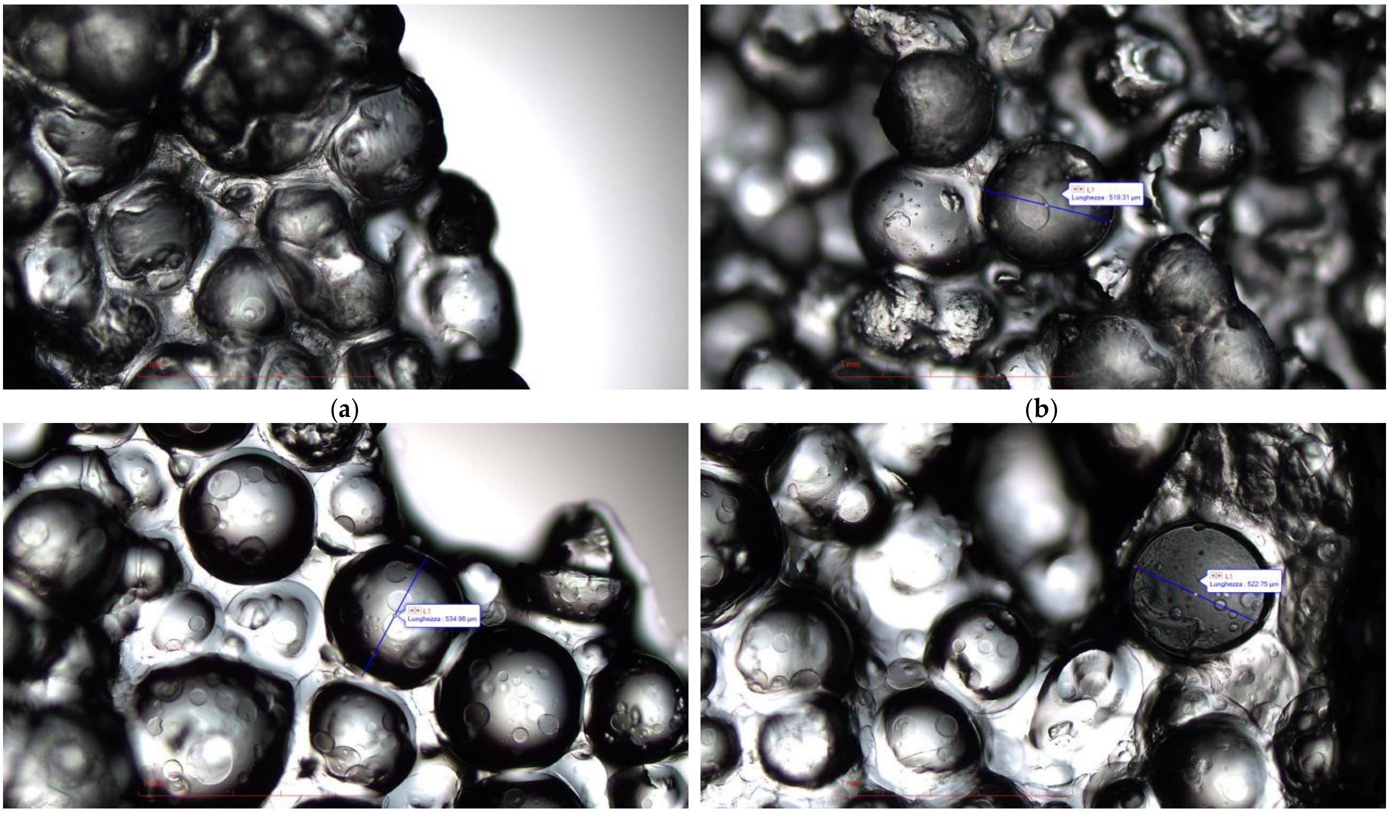

(**a**) (**b**)

(**c**) (**d**)

Figure S5. Optical microphotographs (using a 4 × objective) of R1HG (**a**, **b**) and R2HG (**c**, **d**), at their EDS.

Section S3. Dyes Removal Experiments using R1HG and R2HG.

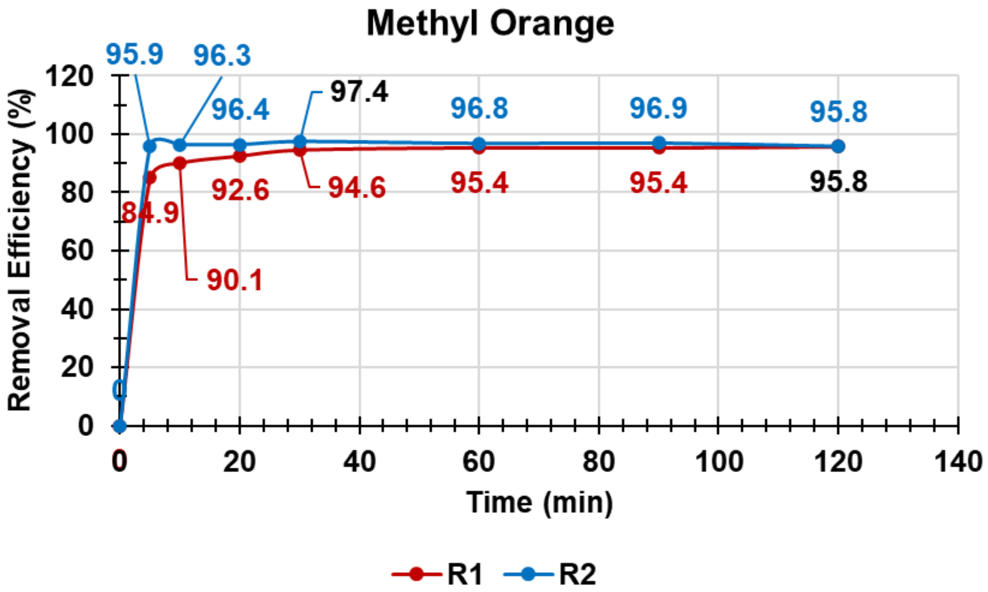

(**a**)

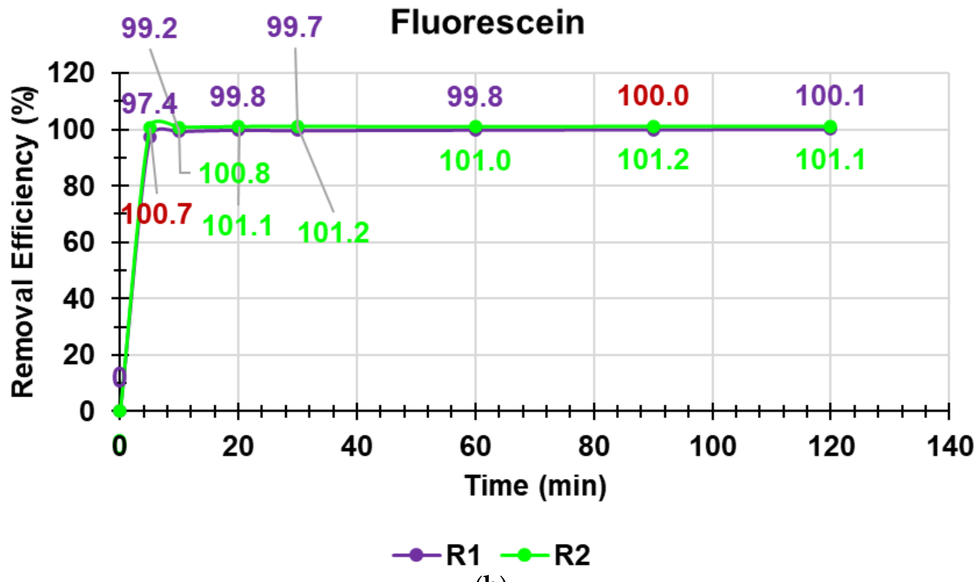
Fluorescein
Removal Efficiency (%)
Time (min)
99.2
99.7
97.4
99.8
99.8
100.0
100.1
100.8
101.0
101.2
101.1
100.7
101.1
101.2
0
R1
R2

(**b**)

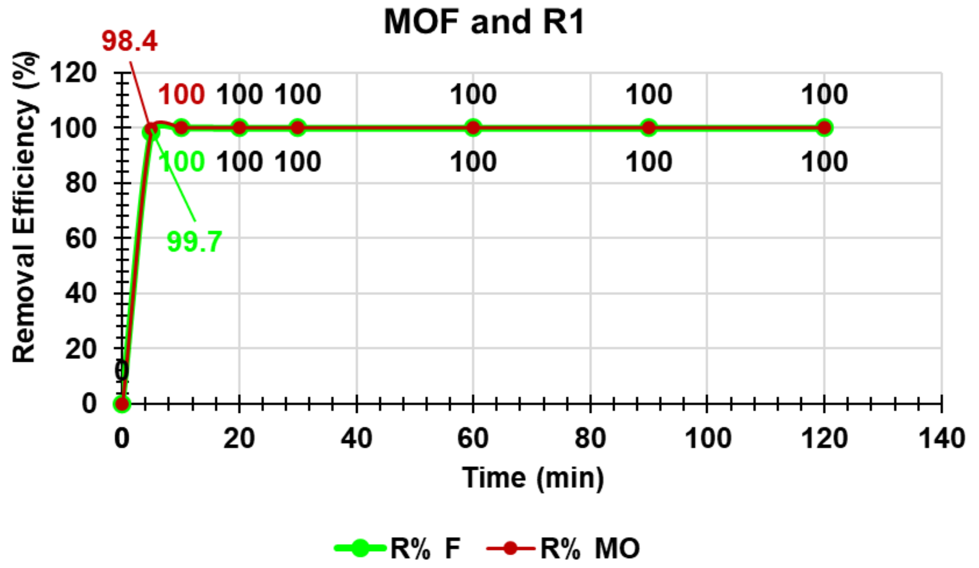
MOF and R1
Removal Efficiency (%)
Time (min)
98.4
100 100 100
100
100
100
100 100 100
100
100
100
99.7
0
R% F
R% MO

(**c**)

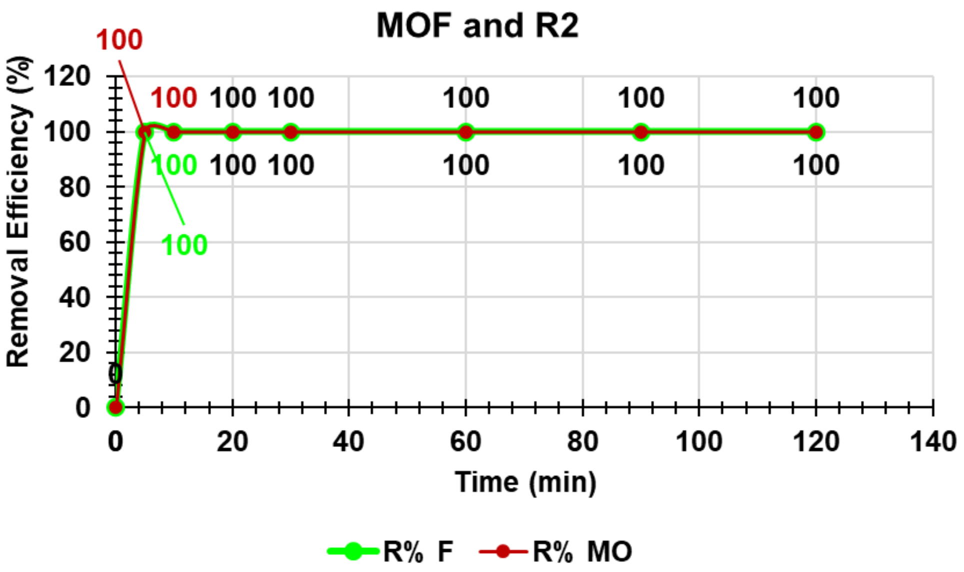

(d)

Figure S6. Cumulative removal efficiency (R%) (pH = 7.5; r. t.) of MO by R1 (red line) and R2 (blue line) (**a**); cumulative removal efficiency (R%) (pH = 7.5; r.t) of F by R1 (purple line) and R2 (green line) (**b**); cumulative removal efficiency (R%) (pH = 7.5; r.t) of MO (red line) + F (green line) (MOF) by R1 (**c**) and by R2 (**d**). F initial concentrations were 48.7 mg/L (R1), 49.4 mg/L (R2), 64.6 mg/L in MOF solution treated with R1, 64.9 mg/L in MOF solution treated with R2. MO initial concentrations were 50.9 mg/L (R1), 50.8 mg/L (R2), 39.6 mg/L in MOF solution treated with R1, 39.7 mg/L in MOF solution treated with R2.

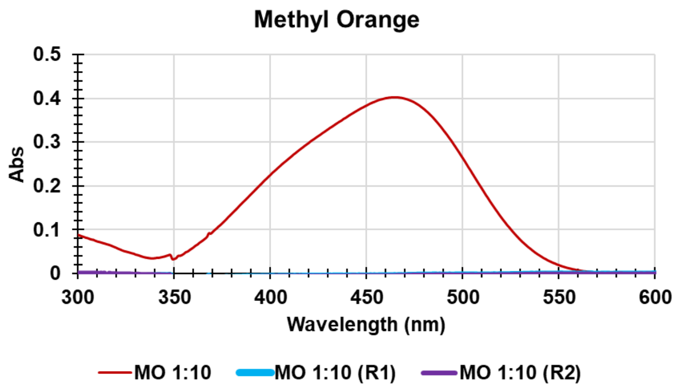

(a)

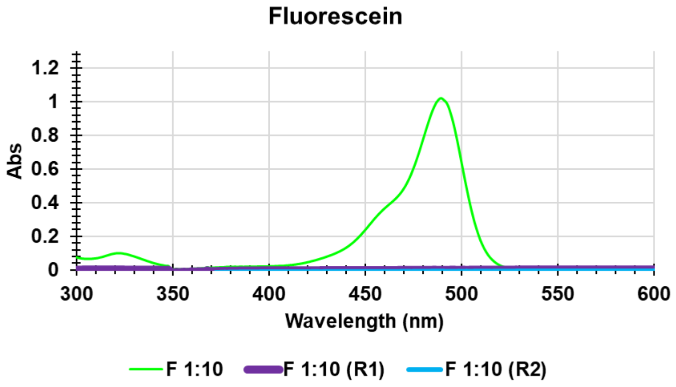

(b)

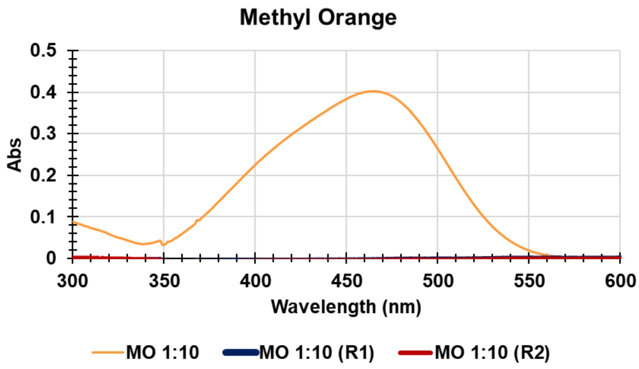

(c)

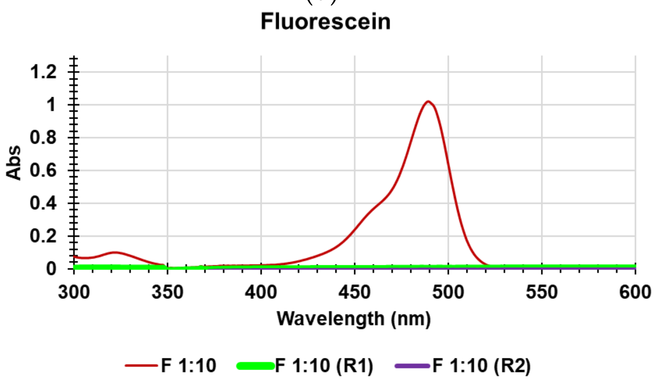

(d)

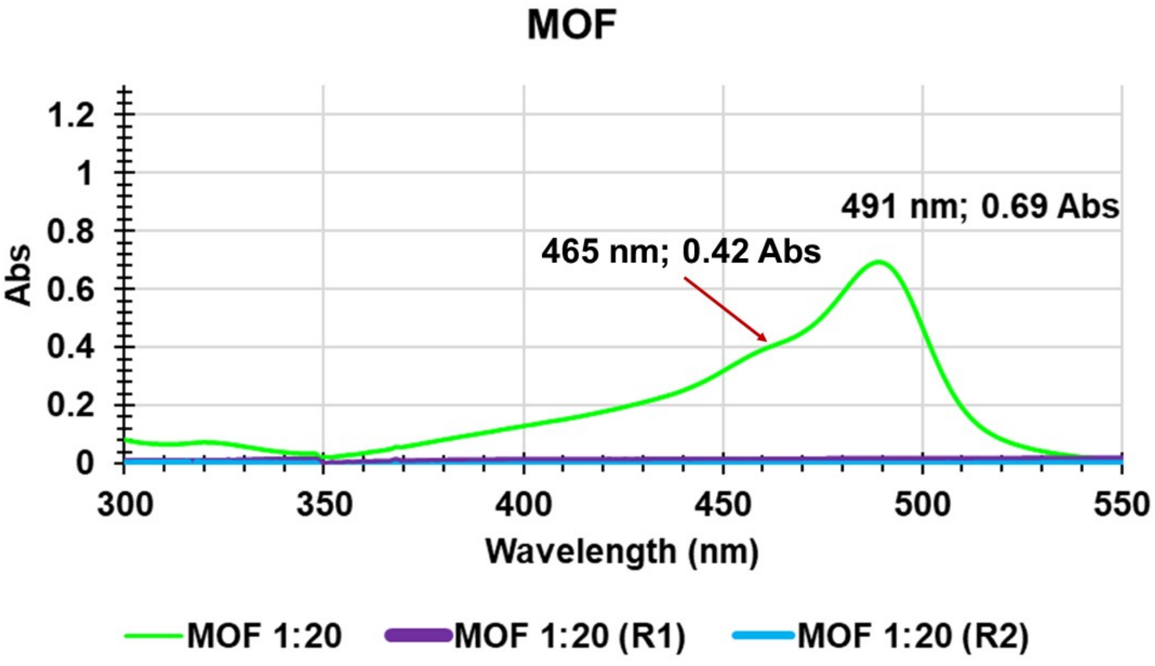

(**e**)

Figure S7. UV-Vis spectra of MO (**a**, **c**), F (**b**, **d**) and MOF (**e**) solutions before filtration (red or green lines) and after filtration through R1HG (purple lines) and R2HG (light blue lines). Figure 14 c, d refer to filtration experiments of MO solution followed by F solution carried out without refreshing the hydrogel. Columns: 8 mL hydrogels; pH = 7.5; r. t. Filtrations were first by gravity and were finished by applying compressed air to the top of columns up to completely empty the columns.

Section S4. Nitrite Removal Experiments using R1HG and R2HG.

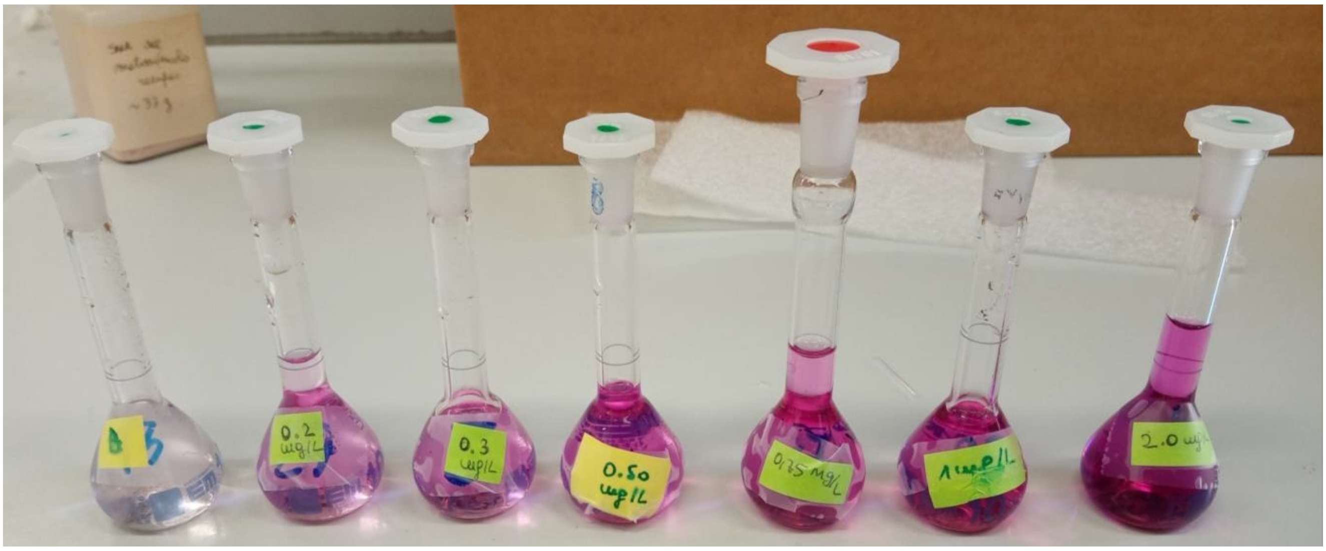

Figure S8. NO_2^- standard solutions (STD1-SDT6) in the concentration range (0.2-2 mg/L; 0.0043-0.0435 mM) and blank (B) reacted with Griess reagent system (GRS) and used to construct the calibration curve shown in Figure S9.

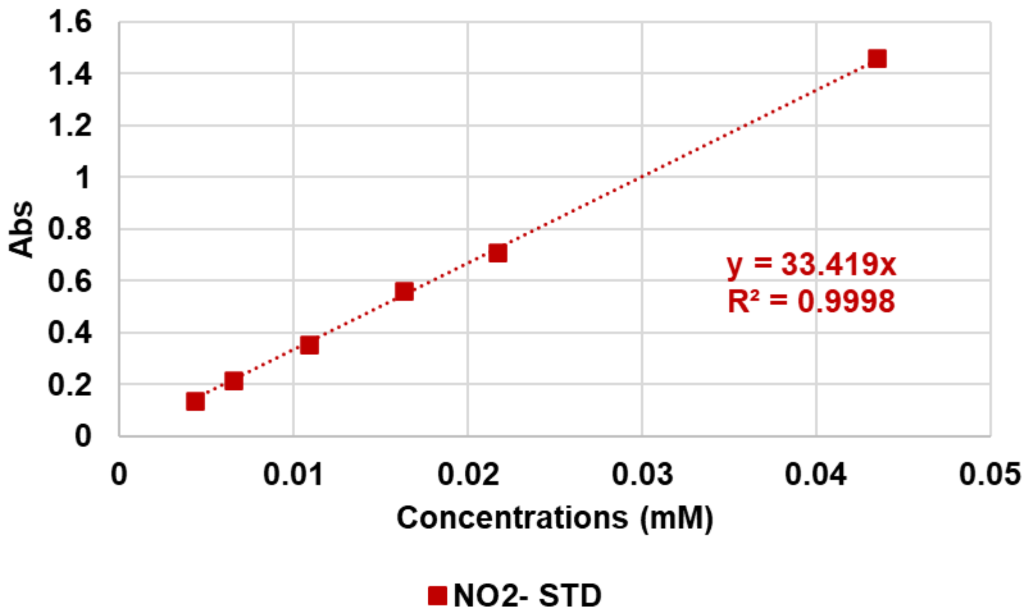

Figure S9. NO_2^-calibration curve in the concentration range 0.0043-0.0435 mM (0.2-2 mg/L).

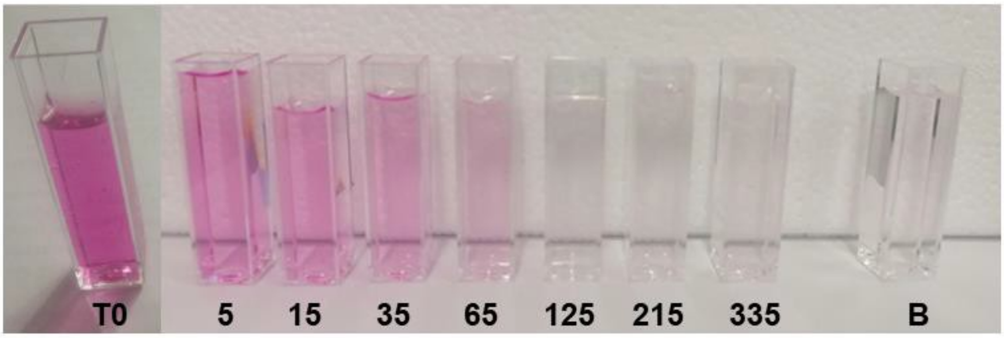

Figure S10. Appearance of NO_2^- solutions treated with the GRS at time T0 (untreated solution, cuvette 1 on the left), of NO_2^- solutions during treatment with R1 after 5, 15, 35, 65, 125, 215 and 335 min (cuvette 2–8 from the left to right) and of the blank solution (cuvette B).

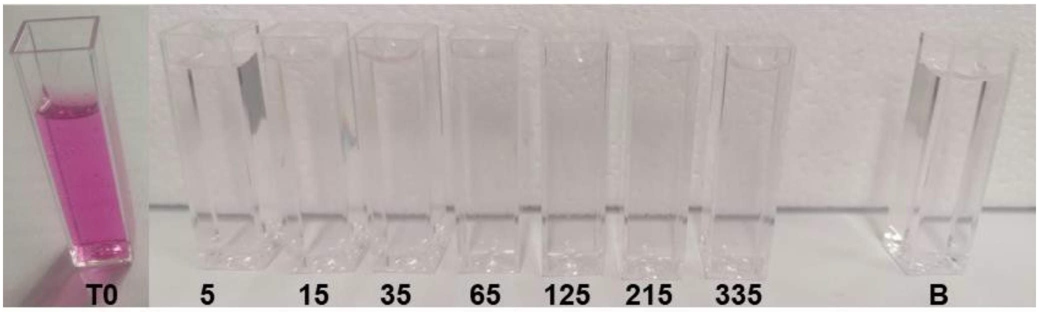

Figure S11. Appearance of NO_2^- solutions treated with the GRS at time T0 (untreated solution, cuvette 1 on the left), of NO_2^- solutions during treatment with R2 after 5, 15, 35, 65, 125, 215 and 335 min (cuvette 2–8 from the left to right) and of the blank solution (cuvette B).

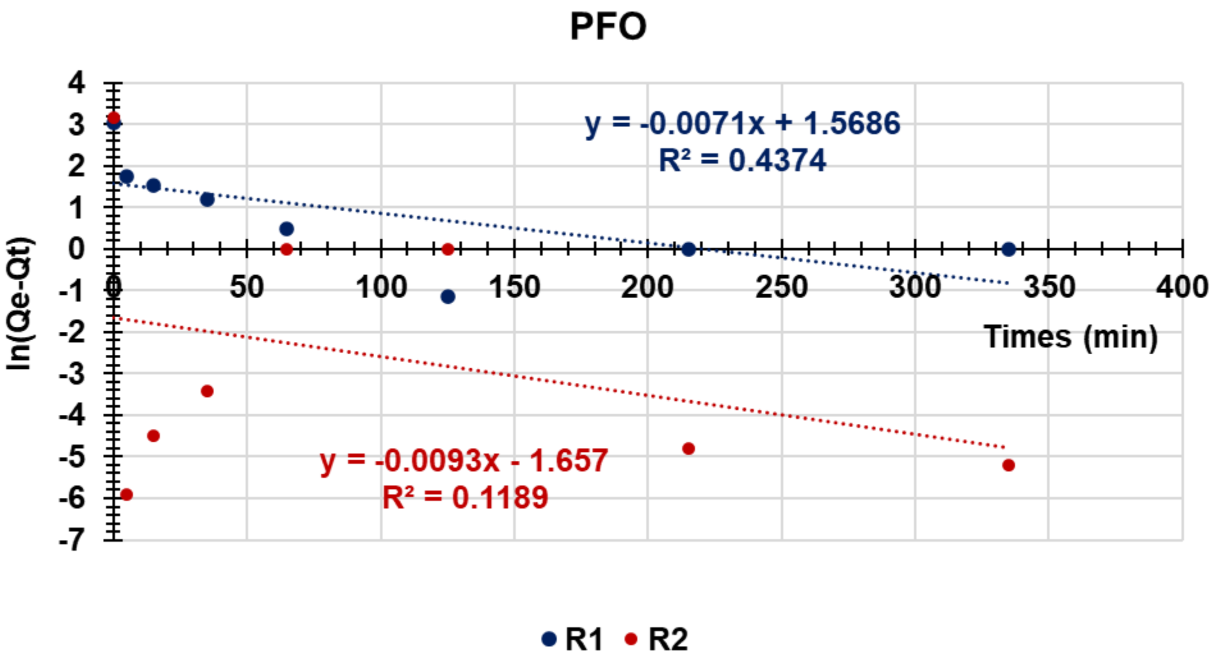

Figure S12. PFO kinetic model (Qe and Qt expressed as mg/g).

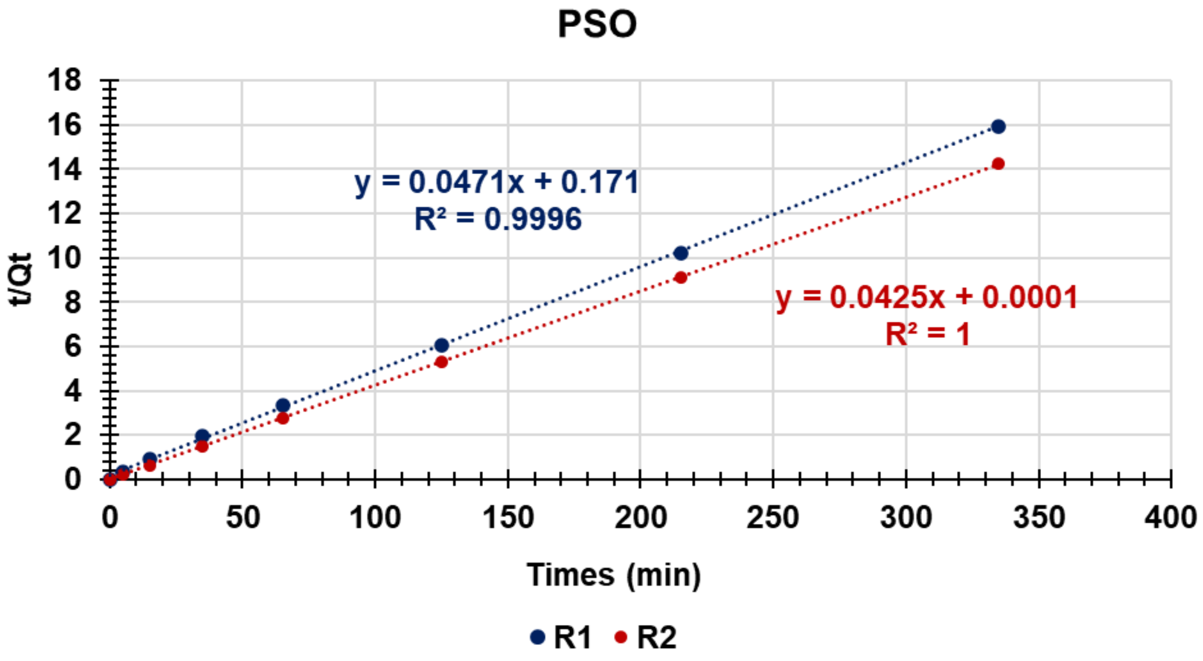

Figure S13. PSO kinetic model (Qt expressed as mg/g; t/Qt = g/mg×min).

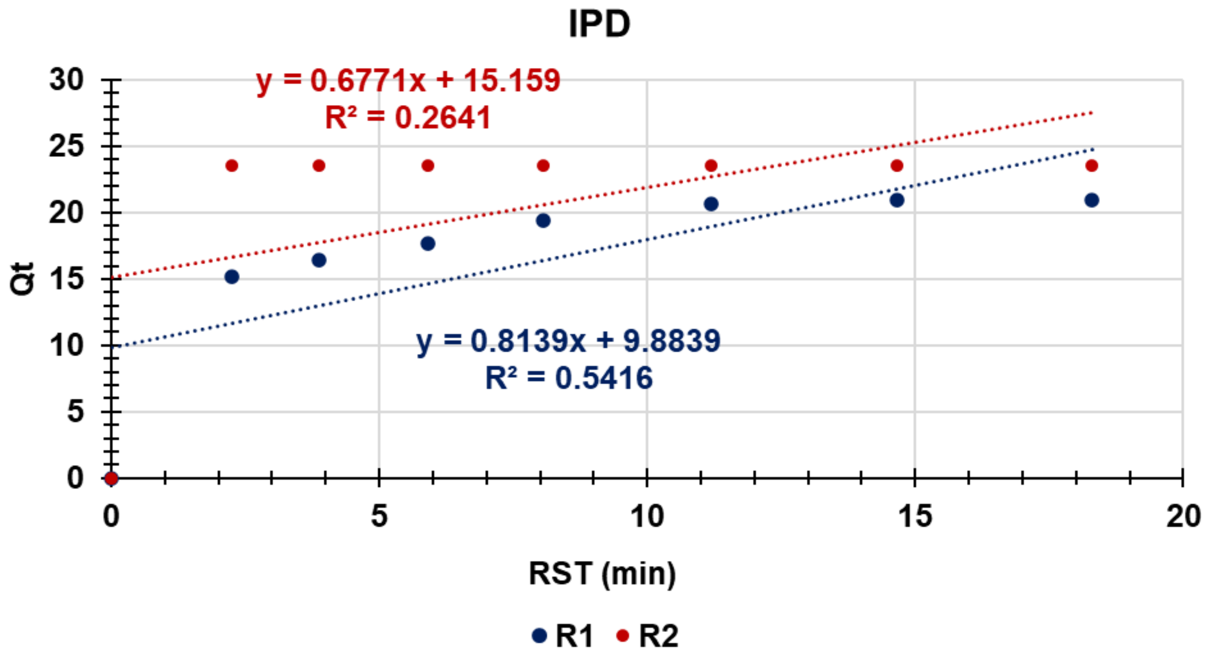

Figure S14. IPD kinetic model (Qt expressed as mg/g). RST = root square of times.

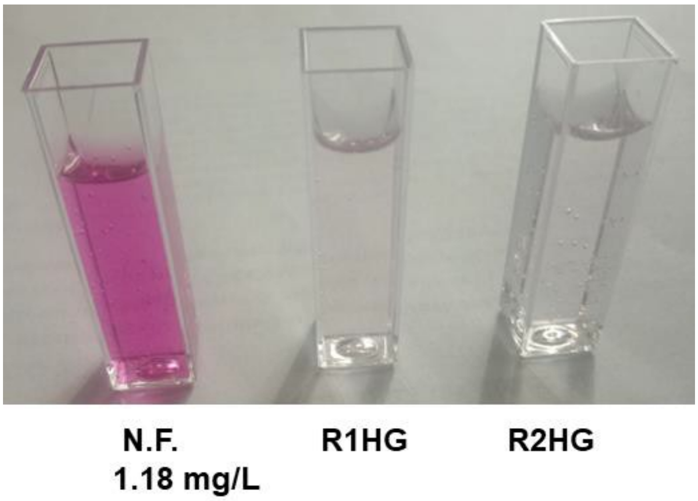

Figure S15. NO_2^- solutions before filtration (N.F.) and after filtration by R1 (from the left second cuvette**)** and by R2 (from the left third cuvette).

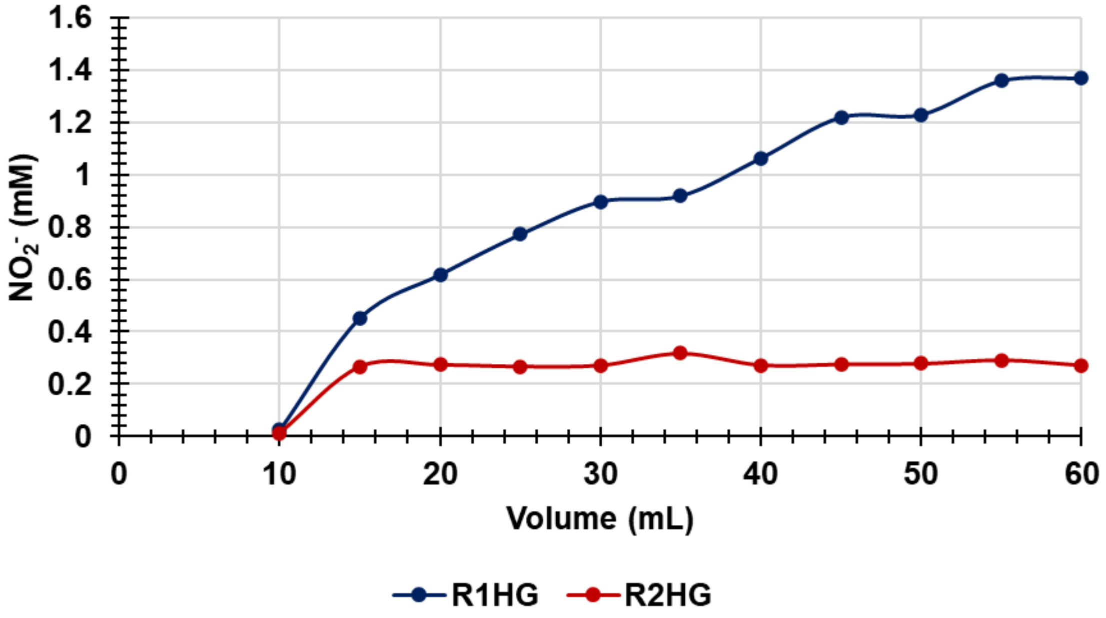

Figure S16. Nitrite concentration in the solutions filtered by R1HG (blue line) and R2HG (red line) as a function of the volumes of NO_2^- solutions 118 mg/L (2.57 mM) sequentially filtered under pressure. Columns: 10 mL hydrogels; pH = 7.5; room temperature (r.t.). Error bars not detectable.
